# Gamification Elements in Physical Activity Interventions: Scoping Review and Taxonomy Based on the Octalysis Framework

**DOI:** 10.2196/85804

**Published:** 2026-09-25

**Authors:** Sijia Li, Li Qiao, Rui-Ning Wang, Runyuan Pei, Hao-Ming Ma, Mei-Hua Piao

**Affiliations:** 1 Chinese Academy of Medical Sciences, Peking Union Medical College School of Nursing, No 33 Ba Da Chu Road, Shijingshan District, Beijing, 100144, China, 86 13522112889; 2 Department of Nursing, West China Hospital/West China School of Nursing, Sichuan University, Chengdu, China

**Keywords:** gamification, physical activity, gamification octalysis framework, behavior change, PRISMA

## Abstract

**Background:**

Physical inactivity affects approximately 1.4 billion adults globally, representing a major public health challenge. Gamification shows potential in addressing global inactivity by boosting motivation. The widely recognized Gamification Octalysis Framework, which emphasizes human motivation over mere functionality, can be used to summarize gamification elements in physical activity (PA) applications. However, previous reviews have mainly examined the effectiveness of gamified PA interventions, with limited attention to how gamification elements are linked to motivational mechanisms and behavior change techniques (BCTs). Additionally, the integration of identified BCTs can help connect game elements and health behavior change.

**Objective:**

This scoping review aimed to map and categorize gamification elements used in PA interventions according to the Octalysis Framework, identify associated BCTs, and describe reported outcome domains and design gaps to inform future gamified PA intervention development.

**Methods:**

The review protocol was prospectively registered with the International Prospective Register of Systematic Reviews (PROSPERO; CRD420251077817). The scoping review was conducted according to the PRISMA-ScR (Preferred Reporting Items for Systematic Reviews and Meta-Analyses extension for Scoping Reviews). A total of seven databases (ie, PubMed, Embase, Cochrane Library, Web of Science, CINAHL, SPORTDiscus, and Psychological Information) were selected and searched for relevant randomized controlled trials (RCTs) published in English from inception to July 7, 2026. Two reviewers (SJL and LQ) independently conducted study selection and gamification-element coding. Data charting was performed by one reviewer (SJL) and verified by another (LQ).

**Results:**

A total of 53 RCTs and 34 unique interventions were included in this review. The studies reported a variety of outcomes, including user retention, behavioral changes, psychological impacts, and health-related indicators. The most frequently adopted BCT elements were “2.2 feedback on behavior” and “1.1 goal setting (behavior).” Moreover, using the Octalysis framework, a taxonomy was developed, identifying 48 gamification elements (eg, narrative, mentoring, and points loss) across 8 core motivational drives. The majority of BCTs were mapped to 2 core motivations (ie, empowerment and accomplishment), while some BCTs were not supported by gamification elements.

**Conclusions:**

This scoping review complements previous effectiveness-focused reviews by examining how gamified PA interventions are designed and which motivational and behavior change functions they target. By developing an Octalysis-based taxonomy of 48 gamification elements and mapping these elements against BCTs, this review provides a descriptive, design-oriented evidence map of commonly used design patterns and underrepresented behavior change functions. The findings may support more deliberate and transparent selection, combination, and reporting of gamification elements in future PA interventions. However, they should not be interpreted as evidence of the effectiveness of individual gamification elements, and further research is needed to evaluate how specific design configurations influence engagement and PA outcomes.

## Introduction

Participating in regular physical activity (PA) offers physical and mental health benefits and helps reduce the risk of noncommunicable diseases [1,2]. According to the World Health Organization (WHO)’s 2020 guidelines on PA, adults should aim for at least 150 minutes of moderate-intensity activity, 75 minutes of vigorous-intensity activity, or an equivalent combination of both [3]. Despite the well-established benefits of PA, approximately one-third of adults do not meet the minimum recommended levels of activity [4]. Physical inactivity contributes significantly to the global health burden, accounting for over 7% of deaths from all causes and cardiovascular diseases, as well as up to 8% of noncommunicable diseases [5]. The global economic cost of inaction on physical inactivity is estimated to reach approximately $47.6 billion annually [6]. Given the health impacts and economic burden of physical inactivity, it is crucial to take immediate action to promote PA.

At the individual level, lack of motivation is the most commonly cited barrier to PA [7]. According to self-determination theory (SDT), sustainable behavior change occurs when interventions support 3 basic psychological needs: autonomy, competence, and relatedness [8]. Gamification, which involves the use of game elements (eg, points, levels, avatars, and leaderboards) into nongame contexts (eg, education, training, and health care), has emerged as a promising strategy for addressing motivational barriers to PA by potentially fulfilling these psychological needs [9]. It can enhance user experiences, motivation, and engagement in PA applications, particularly in the context of rapid technological advancement [10].

However, recent systematic reviews reveal mixed effectiveness of gamification interventions for PA promotion, with studies reporting varying success rates and limited long-term sustainability [11]. This inconsistency may be attributed to the lack of systematic approaches that align game elements with underlying psychological motivations and established behavior change principles. In 2022, Xu et al [12] conducted a systematic review to summarize gamification interventions aimed at increasing PA participation, but the review focused primarily on the game elements without addressing their connection to psychological motivation. Zichermann and Cunningham [13] suggest that gamification is composed of 75% psychology and 25% technology. Research in behavioral psychology demonstrates that effective behavior change interventions must address underlying motivational processes rather than relying solely on superficial game mechanics [14]. This disconnection between game elements and motivational theory represents a fundamental limitation in current gamification approaches for PA promotion.

To address this theoretical gap, frameworks that systematically integrate game elements with psychological motivations are essential. The Gamification Octalysis Framework, introduced by Chou in 2019, is widely applied across various fields and is well-known for prioritizing human motivation over functionality to enhance the effectiveness of gamification [15,16]. The framework builds on established motivational theories, particularly SDT, by organizing game elements according to 8 core psychological drives that influence human behavior and decision-making. His Octalysis Framework consists of 8 core drives that can motivate, empower, or influence behavioral patterns, ultimately leading us to take actions. Given the framework’s potential in combining gamification elements with human motivation, it can be applied to summarize gamification elements in PA applications, providing a reference for future gamification intervention designs that promote PA by altering motivation.

The integration of behavior change techniques (BCTs) can help to better align game elements with health behavior change, fostering a more seamless connection between the two [17,18]. In 2013, Michie et al [19] systematically summarized 93 BCTs, such as habit formation, self-monitoring, and action planning**,** with subsequent updates expanding the taxonomy to include digital health-specific techniques. These techniques represent the minimal, replicable components of an intervention, aimed at influencing or altering the causal processes that regulate behavior, and can be applied to guide the design of behavioral intervention.

Previous reviews have mainly examined whether gamified interventions are effective in increasing PA, but less attention has been paid to how gamification elements are theoretically structured, which motivational drives they target, and how they are integrated with established BCTs [11,12]. This represents a design-level gap because the effectiveness and sustainability of gamified PA interventions may depend not only on the presence of game elements, but also on whether these elements are aligned with motivational mechanisms and behavior change functions. Although scoping reviews may be conducted as precursors to systematic reviews, they can also be used to map the conceptual structure and characteristics of an established evidence base and to identify gaps that are not addressed by existing effectiveness reviews [20]. Therefore, the presence of previous systematic reviews does not eliminate the need to examine how gamified PA interventions are designed and how their components are theoretically organized.

A scoping review approach was appropriate because the primary purpose of this review was to map the conceptual gamification elements used in PA interventions, rather than to estimate pooled intervention effects [21]. The present review was therefore not undertaken to determine whether an additional effectiveness review was warranted, but to address a distinct design-oriented question. Specifically, the scoping review aimed to categorize gamification elements used in PA applications using the Octalysis Framework, identify associated BCTs, and examine how BCTs corresponded to gamification elements and motivational drives. It also aimed to identify motivational and behavior change functions that were underrepresented or not clearly supported by existing gamification elements. This review therefore complements, rather than duplicates, previous effectiveness-focused systematic reviews by providing an Octalysis-based taxonomy and a design-oriented evidence map. These outputs may support more transparent description, selection, and integration of gamification elements according to the intended motivational and behavior change objectives of future PA interventions.

## Methods

### Overview

The scoping review was conducted following the PRISMA-ScR (Preferred Reporting Items for Systematic Reviews and Meta-Analyses extension for Scoping Reviews) [22]. The study was registered on the International Prospective Register of Systematic Reviews (PROSPERO; registration number CRD420251077817). The review was originally registered in PROSPERO as a systematic review. Following editorial guidance and reconsideration of the review objectives, we revised the study as a scoping review because the primary purpose was evidence mapping and taxonomy development rather than pooled effectiveness estimation. This methodological change is reported transparently as a deviation from the original registration.

### Search Strategy

The seven databases (ie, PubMed, Embase, Cochrane library, Web of Science, CINAHL, SPORTDiscus, and PsycINFO) were selected and searched for relevant randomized controlled trials (RCTs) published in English from inception to February 28, 2025. The searches were updated on July 7, 2026, by rerunning the database-specific search strategies in all seven databases. No limits on language, age, sex, health condition, or publication date were applied during the database search. Terms relating to RCTs were incorporated into the search strategies because the review was restricted to interventions evaluated using an RCT design. Relevant previous reviews were consulted to identify potentially relevant terminology; however, no previous search strategy was substantively reused or adapted [11,12]. The search strategy was developed specifically for the objectives of the present review. The systematic search was developed and reported in accordance with the PRISMA-S (Preferred Reporting Items for Systematic Reviews and Meta-Analyses literature search extension) guidelines [23]. The comprehensive search strategy, including all controlled vocabulary (eg, MeSH and the Embase subject headings EMTREE terms), field modifiers, and synonyms used across all seven databases, is detailed in Supplementary Table 1 in Multimedia Appendix 1, and the completed PRISMA-S checklist is provided in Checklist 1. The search strategy was internally reviewed by another member (MHP) of the review team for conceptual relevance, completeness, and database-specific syntax. Although CINAHL, SPORTDiscus, and PsycINFO were accessed through Elton B. Stephens Company host (EBSCOhost; EBSCO Information Services), each database was searched separately; therefore, no simultaneous multidatabase search was performed. No clinical trial registries were searched. In addition, no other online or print sources, such as websites, tables of contents, or conference proceedings, were purposefully searched or browsed. The review focused on published full-text RCT reports because sufficiently detailed descriptions of intervention components were required for the reliable coding of gamification elements and BCTs.

Backward and forward citation searching was conducted for all included RCT reports. For backward citation searching, the reference lists of these articles were examined. For forward citation searching, articles citing these reports were identified using Web of Science Core Collection and OpenAlex. Citation searching was completed on July 21, 2026. All newly identified records were deduplicated and screened according to the same eligibility criteria applied during the main database screening process. No additional search methods were used beyond database searching and backward and forward citation searching. Authors were contacted through ResearchGate to request full-text articles that could not otherwise be accessed.

### Study Selection and Eligibility Criteria

All retrieved studies were imported into EndNote 21 (Clarivate). Duplicate records were identified using the find duplicates function in EndNote. The deduplicated records were exported to the Rayyan website (Rayyan Systems, Inc.) for the study selection process [24]. Two authors (SJL and RNW) independently screened the titles and abstracts of the retrieved studies, and the full texts of potentially eligible studies were reviewed to finalize the included studies. Any disagreements were resolved by a third author (QL).

Articles were considered eligible if they met the following criteria according to the PCC framework (Population, Concept, Context):

1. Population: Any human population, regardless of age or health status (no age- or condition-specific population terms were applied in the search syntax. The population component was intentionally left open to maximize sensitivity and to capture gamified PA interventions involving any human population);
2. Concept: gamification elements used as motivational features in PA interventions;
3. Context: PA interventions delivered through digital applications.

Conference papers and full-text articles with missing or inaccessible extracted data were excluded. Only full-text reports published in English were eligible for inclusion.

### Data Charting Process

Data were charted using a standardized Excel (Microsoft)-based form developed according to the review objectives and the prespecified data items. The form was initially tested on a subset of included studies and refined to improve clarity and consistency before full data charting. One reviewer (SJL) charted the data, and a second reviewer (QL) verified all charted information. Any discrepancies were resolved through discussion and, when necessary, consultation with a third reviewer (MHP). When multiple reports described the same intervention, the reports were treated as one unique intervention for the analysis of BCTs and gamification elements, while study characteristics and outcomes were charted separately for each report. Information was obtained from the main articles and, where available, associated protocols, supplementary materials, intervention screenshots, and other reported intervention materials. When relevant information remained unclear or unavailable, the study authors were contacted for clarification or access to additional materials.

### Data Items

Articles information (ie, author, year, country, and study arms), population characteristics (including health status, sample sizes, average age, and sex distribution), intervention and control descriptions, primary and secondary outcomes, and study conclusions were charted. To provide a comprehensive understanding of behavior change interventions using gamification strategies, the following information was also charted:

- BCTs: identified according to Michie’s BCT Taxonomy (BCTTv1; Supplementary Table 2 in Multimedia Appendix 2) [19];
- Gamification elements: summarized based on the Octalysis Framework proposed by Chou (Figure S1 in Multimedia Appendix 3) [16].

### Taxonomic Development

#### Framework Selection and Justification

We selected the Octalysis Framework for taxonomic development because it provides a motivation-oriented structure for categorizing gamification elements according to 8 core drives, making it suitable for examining how game elements are used to support PA behavior change [16]. Other frameworks, such as the Gameful Design Heuristics proposed by Tondello et al [25], also provide valuable guidance for gameful design. However, this framework was developed mainly as an inspection tool for evaluating the design quality and motivational affordances of existing gameful systems, rather than as a taxonomy for classifying intervention components. The Gameful Design Heuristics include 28 heuristics for rapidly evaluating gameful systems. Therefore, although useful for assessing gameful design, it was less aligned with the aim of this review, which was to map gamification elements, classify them according to motivational drives, and compare them with BCTs.

#### Coding Development and Gamification Element Identification

The coding of gamification elements was performed according to the existing definitions of motivational drives in the Octalysis framework. Two independent reviewers (SJL and QL), both with expertise in health behavior change, coded all gamification elements from article intervention descriptions, application screenshots, supplementary materials, and protocols. The coding focused on identifying the psychological drives each element aims to drive. Elements serving multiple drives were categorized based on their primary psychological mechanism. Any discrepancies in the process were resolved by discussion with the third author (MHP).

### Synthesis of Results

Charted data were synthesized using descriptive statistics and qualitative conceptual mapping. Basic study characteristics, intervention descriptions, and diverse outcome domains were summarized narratively and presented in tabular formats. To address the primary objectives and construct the taxonomy, gamification elements were categorized into the 8 core drives of the Octalysis Framework, and identified BCTs were aggregated by frequency. Finally, a cross-mapping analysis between the BCTs and gamification elements was conducted. Data visualizations, including evidence gap maps, were generated to illustrate research concentrations and explicitly identify behavioral strategies currently lacking corresponding gamification support.

## Results

### Selection of Sources of Evidence

The complete literature selection process is illustrated in Figure 1. A total of 9242 records were identified through database searches. After removing 3929 duplicates using EndNote 21, the titles and abstracts of 5313 studies were screened. Among these, 5225 records were excluded for failing to meet the eligibility criteria, leaving 88 studies for full-text review. Following this, 35 records were excluded due to wrong study design (n=2), wrong intervention (n=24), wrong outcome (n=6), duplicates (n=2), and full-text unavailable (n=1; reasons documented in Table S3 in Multimedia Appendix 4). Ultimately, 53 RCTs were included in this review [26-78].

**Figure 1.**
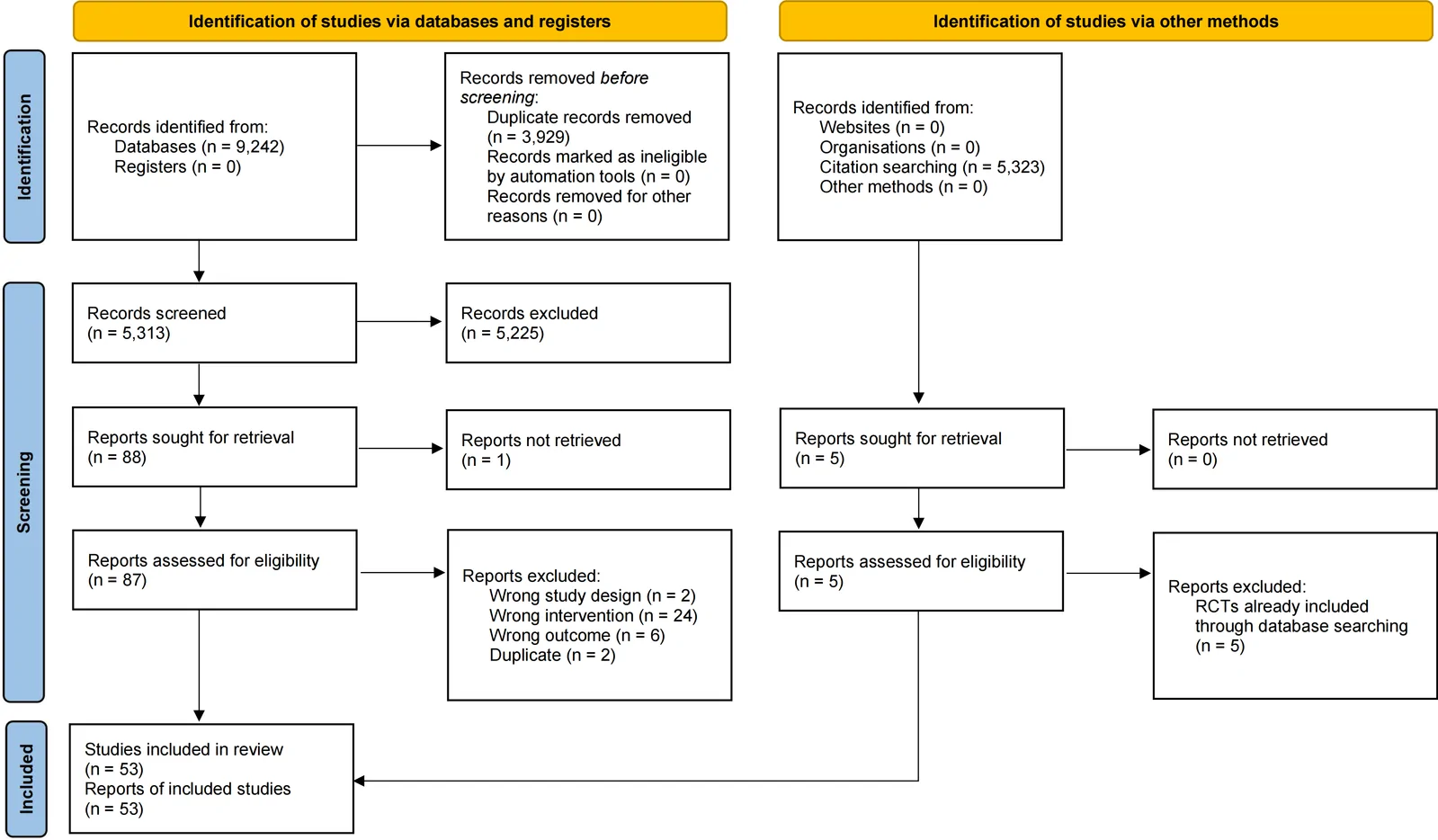
PRISMA-ScR (Preferred Reporting Items for Systematic Reviews and Meta-Analyses extension for Scoping Reviews) flow diagram illustrating the literature search and study selection process for the scoping review. RCT: randomized controlled trial.

Backward citation searching identified 2682 records, and forward citation searching identified 2641 records, resulting in 5323 records in total. After 1803 duplicate records were removed, 3520 unique records remained. A further 110 records had already been identified and screened through the database searches, leaving 3410 additional records for screening. Of these, 925 review articles were excluded because they did not meet the eligible study design criterion, leaving 2485 records for further screening. Finally, no additional eligible RCTs were identified through backward or forward citation searching.

### Characteristics of Sources of Evidence

The characteristics of the 53 included studies are summarized in Table 1. The RCTs were published between 2014 and 2026. Among them, 20 studies (20/53, 37.74%) were conducted in the USA, 17 (17/53, 32.08%) in European countries, 9 (9/53, 16.98%) in Asia, 2 (2/53, 3.77%) in Canada, 2 (2/53, 3.77%) in Australia, 1 (1/53, 1.89%) in New Zealand, and 2 (2/53, 3.77%) in Brazil. Most of the studies (n=40) implemented a 2-arm RCT design, while 8 studies used a 3-arm design, 4 used a 4-arm design, and 1 study used a 5-arm RCT design. The included studies encompassed a diverse range of populations, such as insufficiently active adults, cancer survivors, and individuals who were overweight or obese. Sample sizes varied from 12 to 1062 participants, and mean participant ages ranged from 4.9 to 70.4 years. Two studies included only male participants, while one study included only female participants [45,46,57].

**Table 1.** Baseline characteristics, geographical distribution, population types (including health status), and study designs of the 53 randomized controlled trials evaluating gamified physical activity interventions.

Author and Year	Country	Study arms	Population type	Sample size (IG/CG)	Average age (years)	Sex difference (Male/female)	Control contents
Agarwal et al 2021 [26]	United States	3-arm RCT^a^; IG^b^1: gamification with social support IG2: gamification with social support and loss-framed financial incentives. CG^c^: feedback only.	Veterans with obesity or overweight.	IG1: 60 IG2: 58 CG: 60	IG1: 53.7 (13) IG2: 57.9 (12.8) CG: 58.1 (12.6)	IG1: 31/29 IG2: 40/20 CG: 32/22	Received feedback and goal-setting from the device.
Corepal et al 2019 [27]	Ireland	2-arm RCT IG: the StepSmart Challenge CG: no intervention control group.	Year 9 school children (aged between 12 to 14 years).	IG: 126 CG: 64	—^d^	IG: 48/94 CG: 57/25	No intervention
Dadaczynski et al 2017 [28]	Germany	2-arm RCT IG: Healingo Fit CG: no intervention control group.	Employees of an automobile manufacturer.	IG: 80 CG: 64	—	IG: 49/31 CG: 44/20	No intervention
Direito et al 2015 [29]	New Zealand	3-arm RCT IG1: immersive app (Zombies, Run) IG2: nonimmersive app (Get Running) CG: usual behavior	Young people aged 14 to 17 years.	IG1: 17 IG2: 16 CG: 18	IG1: 15.78 (1.11) IG2: 15.69 (1.04) CG: 15.55 (1.32)	IG1: 8/9 IG2: 6/10 CG: 8/10	No intervention
Edney et al 2019 [30]	Australia	2-arm RCT IG1: gamified version of app IG2: nongamified version of app.	Participants aged 18 to 65 years	IG1: 141 IG2: 160	IG1: 43.3 (11.5) IG2: 40.4 (12.2)	IG1: 35/106 IG2: 44/116	—
Alexander et al 2024 [31]	United States	4-arm RCT IG1: behaviorally designed gamification IG2: loss-framed financial incentives IG3: gamification+financial incentives CG: attention control	Patients at high risk of cardiovascular events	IG1: 304 IG2: 302 IG3: 305 CG: 151	IG1: 67.2 (8) IG2: 66.4 (8.2) IG3: 66.6 (8.2) CG: 66.6 (8)	IG1: 107/197 IG2: 129/173 IG3: 130/175 CG: 54/97	Received a text message for feedback.
Francis et al 2021 [32]	United States	2-arm RCT IG: Fitbit plus MapTrek CG: Fitbit alone.	Individuals predisposed to type 2 diabetes.	IG: 196 CG: 192	IG: 46.9 (13.2) CG: 45.8 (13.8)	IG: 44/152 CG: 45/147	Provided with a Fitbit activity monitor.
Grade et al 2016 [34]	Canada	2-arm crossover RCT IG: MKMM^e^ CG: wear the activity monitor only.	Healthy students.	IG: 28 CG: 28	IG: 11.36 (1.20) CG: 11.36 (1.20)	IG: 26/16 CG: 26/16	Provided with an activity monitor and received daily activity feedback.
Garde et al 2018 [33]	Canada	2-arm RCT IG: MKMM CG: wear the activity monitor only.	Elementary school students.	IG: 19 CG: 18	IG: 10.6 (0.56) CG: 10.6 (0.48)	IG: 8/11 CG: 8/10	Provided with an activity monitor.
Gillcrist et al 2024 [35]	United States	2-arm RCT IG: game-based intervention CG: received weekly updates.	Veterans with knee osteoarthritis.	IG: 16 CG: 15	IG: 60.7 (6.9) CG: 58.4 (18.2)	IG: 14/2 CG: 14/1	Received weekly updates.
Gonze et al 2020 [36]	Brazil	3-arm RCT IG1: smartphone app IG2: smartphone app+tailored messages IG3: smartphone+tailored messages+gamification CG: usual routine	Insufficiently active adults.	IG: 7 CG: 5	IG: 44 (7) CG: 42 (7)	IG: 4/3 CG: 4/1	No intervention.
Gremaud et al 2018 [37]	United States	2-arm RCT IG: Fitbit plus MapTrek CG: Fitbit alone.	Sedentary office workers.	IG: 72 CG: 72	IG: 40.6 (11.7) CG: 40.3 (11.1)	IG: 15/57 CG: 19/53	Provided with a Fitbit activity monitor.
Greysen et al 2021 [38]	United States	2-arm RCT IG: game informed by behavioral economics CG: feedback from wearable device.	Patients discharged from hospital.	IG: 114 CG: 118	IG: 39.7 (15), CG: 40.4 (14)	IG: 46/68 CG: 45/73	Received feedback from wearable device.
Greysen et al 2024 [39]	United States	2-arm RCT IG: game informed by behavioral economics CG: set step goals and received daily feedback.	Older adults at risk for Alzheimer’s disease.	IG: 44 CG: 50	IG: 70.2 (2.9) CG: 70.6 (3.1)	IG: 7/37 CG: 14/36	Set step goals and received daily feedback.
Haque et al 2020 [40]	Finland	2-arm RCT IG: mHealth^f^ app for PA motivation CG: paper diary.	Office-based employees.	IG: 20 CG: 7	—	IG: 10/10 CG: 4/3	Used a paper diary.
Hochsmann et al 2019 [42]	Switzerland	2-arm RCT IG: novel PA^g^-promoting smartphone game CG: one-time lifestyle counseling.	Inactive, overweight, and type 2 diabetes patients (45‐70 years of age).	IG: 18 CG: 17	IG: 56 (5) CG: 58 (6)	IG: 10/8 CG: 9/9	Received one-time lifestyle counseling and structured exercise plan.
Hochsmann et al 2019 [41]	Switzerland	2-arm RCT IG: novel PA-promoting smartphone game CG: one-time lifestyle counseling	Overweight and type 2 diabetes patients (45‐70 years of age)	IG: 18 CG: 17	IG: 56 (5) CG: 58 (6)	IG: 10/8 CG: 9/9	Received one-time lifestyle counseling and structured exercise plan.
Kurtman et al 2018 [43]	United States	3-arm RCT IG1: gamification intervention IG2: gamification intervention with PCP data sharing CG: no intervention.	Obese adults.	IG1: 66 IG2: 64 CG: 66	IG1: 42.3 (11.5) IG2: 39.3 (10.6) CG: 42.5 (12.9)	IG1: 11/55 IG2: 12/52 CG: 5/61	No intervention.
Lee et al 2022 [44]	United States	2-arm RCT IG: a gamified app, Puzzle Walk CG: a commercial app, Google Fit.	Adults with autism spectrum disorder.	IG: 12 CG: 12	IG: 27.1 (7.5) CG: 31.9 (11.3)	IG: 3/9 CG: 6/6	Google Fit app.
Leinonen et al 2017 [45]	Finland	2-arm RCT IG: a gamified web-based mobile service with a wrist-worn physical activity monitor CG: a simple activity monitor.	Young men.	IG: 187 CG: 167	IG: 17.9 (0.7) CG: 17.8 (0.6)	IG: 187/0 CG: 167/0	A simple PA monitor.
Lewey et al 2022 [46]	United States	2-arm RCT IG: gamification intervention CG: received daily feedback.	Postpartum individuals with hypertensive disorder of pregnancy.	IG: 63 CG: 64	IG: 32.7 (5.6) CG: 32.0 (5.6)	IG: 0/63 CG: 0/64	Received wearable device and daily feedback.
Lin et al 2021 [47]	Taiwan	3-arm RCT IG1: mHealth application-COOL^h^ Passport IG2: health promotion cloud system and game-based interactive platform with COOL Passport CG: standard-care.	Youth with congenital heart disease.	IG1: 49 IG2: 47 CG: 47	IG1: 19.20 (2.72) IG2: 19.48 (2.99) CG: 19.74 (2.94)	IG1: 19/30 IG2: 19/28 CG: 18/29	Standard care
Maher et al 2015 [48]	Australia	2-arm RCT IG: online social networking physical activity intervention CG: wait-list control	Insufficiently active adults.	IG: 51 CG: 59	—	IG: 14/37 CG: 12/47	No intervention.
Mamede et al 2021 [49]	Netherlands	2-arm RCT IG: gamified digital app CG: active control.	Office worker.	IG: 118 CG: 116	IG: 47.5 (9.6) CG: 45.9 (10.2)	IG: 55/63 CG: 33/83	A basic version of the app.
Monroe et al 2023 [50]	United States	2-arm RCT IG: technology-delivered PA intervention (TECH)^i^+gamification CG: TECH	Insufficiently active adults.	IG: 57 CG: 59	IG: 40.60 (11.93) CG: 39.69 (8.55)	IG: 12/45 CG: 13/46	TECH app.
Nishiwaki et al 2014 [51]	Japan	2-arm crossover RCT IG: game intervention CG: normal activity monitor	Healthy volunteers.	IG: 20 CG: 20	IG: 31 (3) CG: 31 (3)	IG: 6/14 CG: 6/14	Normal activity monitor.
Nuijten et al 2022 [52]	Netherlands	2-arm RCT IG: personalized complexity parameters CG: general complexity parameters	Government staff members.	IG: 23 CG: 22	—	IG: 5/18 CG: 7/15	Same as the intervention content except general goals.
Patel et al 2017 [54]	United States	2-arm RCT IG: gamification intervention plus daily feedback CG: daily feedback control	Families from the Framingham Heart Study cohort.	IG: 98 CG: 102	IG: 55.7 (9.7) CG: 56.2 (10.1)	IG: 46/52 CG: 42/60	Received daily feedback.
Patel et al 2019 [55]	United States	4-arm RCT IG1: support gamification IG2: collaboration gamification IG3: competition gamification CG: regular feedback control	Overweight and obese adults.	IG1: 151 IG2: 150 IG3: 150 CG: 151	IG1: 39.4 (10.1) IG2: 38.3 (10) IG3: 38.7 (10) CG: 38.5 (10.9)	IG1: 110/41 IG2: 107/43 IG3: 113/37 CG: 97/54	Received regular feedback.
Patel et al 2021 [53]	United States	5-arm RCT IG1: gamification with assigned and immediate goals IG2: gamification with assigned and gradual goals IG3: gamification with choice and immediate goals IG4: gamification with choice and gradual goals CG: daily feedback control	Disadvantaged adults at elevated risk for major adverse cardiovascular events.	IG1: 99 IG2: 99 IG3: 100 IG4: 106 CG: 96	IG1: 57.5 (10.8) IG2: 57.4 (10.6) IG3: 58 (10.9) IG4: 58.7 (11.8) CG: 61.1 (9.3)	IG1: 21/78 IG2: 35/64 IG3: 39/61 IG4: 33/73 CG: 24/72	Received daily feedback.
Patel et al 2021 [56]	United States	4-arm RCT IG1: support gamification IG2: collaboration gamification IG3: competition gamification CG: goal setting and regular feedback control	Adults with uncontrolled diabetes.	IG1: 92 IG2: 95 IG3: 87 CG: 87	IG1: 52.8 (10.4) IG2: 52.5 (9.1) IG3: 51.3 (10.6) CG: 53.4 (10.6)	IG1: 36/56 IG2: 38/57 IG3: 46/41 CG: 39/48	Received regular feedback.
Pyky et al 2017 [57]	Finland	2-arm RCT IG: MOPOrtal service CG: received wearable device	Young adolescent men.	IG: 250 CG: 246	IG: 17.9 (0.7) CG: 17.8 (0.6)	IG: 250/0 CG: 246/0	Received activity monitor.
Tu et al 2018 [65]	China	2-arm RCT IG1: Walkup app IG2: WeChat Sports app	Undergraduate students.	IG: 63 CG: 65	—	—	—
Robertson et al 2018 [58]	United Kingdom	2-arm RCT IG: FitQuest app CG: standard PE lesson	10 to 11 years old from primary school.	IG: 111 CG: 104	—	IG: 55/56 CG: 45/59	Traditional PE lesson.
Robertson et al 2020 [59]	United States	2-arm RCT IG: multimedia messaging service messages plus activity tracker CG: received physical activity tracker	Cancer survivors.	IG: 37 CG: 38	Mean (SD): 55.1 (13.5)	IG: 3/36 CG: 4/35	Provided with a Fitbit activity monitor.
Santos et al 2021 [60]	Japan	2-arm RCT IG: social pervasive game CG: pervasive game without social interaction.	Japanese older adults.	IG: 9 CG: 9	IG: 62.2 (25.2) CG: 64.6 (26.7)	IG: 2/7 CG: 2/7	Pervasive game without social interaction.
Sanudo et al 2024 [61]	Spain	2-arm RCT IG: gamified mHealth intervention CG: control condition without gamification	Young adults.	IG: 39 CG: 37	IG: 21.5 (1.9) CG: 21.7 (1.8)	IG: 17/22 CG: 18/19	Same as the intervention content except social comparison elements.
Schwarz et al 2021 [62]	Netherlands	2-arm RCT IG: dynamically tailored exergame CG: nontailored exergame	Adolescent.	IG: 42 CG: 52	14.61 (1.93)	IG: 28/14 CG: 33/19	Same as the intervention content except tailored feedback.
Simmering et al 2025 [63]	United States	2-arm RCT IG: VA^j^ MapTrek CG: received a Fitbit activity tracker	U.S. Veterans.	IG: 153 CG: 123	IG: 59.9 (12.7) CG: 61.9 (13)	IG: 130/23 CG: 111/12	Provided with a Fitbit activity monitor.
Thorsteinsen et al 2014 [64]	Norway	2-arm RCT IG: lifestyle intervention CG: no intervention	Healthy adults.	IG: 12 CG: 8	Mean (SD): 55.3 (11.2)	11/10	No intervention.
Waddell et al 2022 [66]	United States	2-arm RCT IG: gamification with social incentives CG: received wearable device feedback	Adults with stroke.	IG: 17 CG: 17	IG: 57 (13.8) CG: 61 (16.9)	IG: 6/11 CG: 6/11	Received wearable device feedback.
Xu et al 2024 [67]	China	3-arm RCT IG1: individual gamified intervention IG2: team gamified intervention CG: received daily goal setting	Patients with coronary heart disease.	IG1: 32 IG2: 31 CG: 30	IG1: 52.7 (10.8) IG2: 52.6 (10.8) CG: 53.7 (10.2)	IG1: 28/8 IG2: 30/6 CG: 30/6	Received daily goal setting.
Zuckerman et al 2014 [68]	Israel	3-arm RCT IG1: points version of StepByStep IG2: leaderboard version CG: quantified version	Undergraduate communications Students.	IG1: 21 IG2: 20 CG: 18	Mean (SD): 23.4 (1.4)	15/44	A basic version of the app.
Maria do et al 2026 [75]	Brazil	3-arm RCT IG1: smartphone app IG2: smartphone app+tailored messages IG3: smartphone+tailored messages+gamification CG: usual routine	Insufficiently active adults.	IG: 7 CG: 5	IG: 44 (7) CG: 42 (7)	IG: 4/3 CG: 4/1	No intervention.
Patel et al 2025 [69]	United States	2-arm RCT IG: gamification+automated coaching CG: attention control	Patients with peripheral artery disease.	IG: 51 CG: 52	IG: 69.4 (8.2) CG: 70.0 (9.1)	IG: 29/22 CG: 25/27	Received a wearable device and a daily text message indicating whether the previous day’s step goal was met.
Patel et al 2025 [70]	United States	2-arm RCT IG: behaviorally designed gamification CG: attention control	Black and Hispanic breast and prostate cancer survivors exposed to cardiotoxic therapy and with ≥1 cardiovascular risk factor.	IG: 74 CG: 76	IG: 64.3 (9.3) CG: 63.8 (10.0)	IG: 11/63 CG: 17/59	Received a wearable device and a daily text message reporting the previous day’s step count.
Janis et al 2025 [71]	Germany	2-arm cluster RCT IG: SMARTFAMILY2.0 app CG: waiting-list control	Families with at least 1 adult caregiver and 1 child (>10 years); generally healthy.	Participants IG: 104 CG: 93 (Families IG: 27 CG: 25)	IG adults: female 46.7 (4.98) male 45.0 (5.11) IG children: female 12.1 (3.63), male 11.4 (2.73) CG adults: female 46.4 (6.09), male 45.1 (5.92) CG children: female 10.4 (2.58), male 11.5 (3.64).	IG: 55/49 CG: 48/45	No intervention.
Nimet et al 2025 [72]	Türkiye	2-arm single-blind cluster RCT IG: gamified T2M^k^ android mobile application CG: nongamified T2M android mobile application	Sedentary 7th-grade secondary school students aged 12‐14 years.	IG: 12 CG: 13	12‐14 (range; mean not reported)	IG: 8 girls/4 boys CG: 9 girls/4 boys	T2M mobile application with the same daily 10,000-step and exercise goals, but without gamification elements.
Gaizka et al 2025 [73]	Spain	2-arm single-center pragmatic RCT IG: gamified family-based online exercise platform CG: routine care	Preschool children and their families.	Randomized IG: 40 CG: 40 (Included in per-protocol analysis IG: 32 CG: 39).	IG: 5.1 (0.5) CG: 4.9 (0.5)	IG: 18 boys/22 girls CG: 26 boys/14 girls	Routine care following WHO^l^ recommendations, including regular physical education at school and opportunities for free play with friends in parks or backyards.
Jeffrey Z et al 2026 [76]	Taiwan	4-arm parallel RCT SG: sequential delivery of gamification elements CG: cumulative delivery EG: explicit gamification through Pikmin Bloom; control: tracking only	Healthy university students aged 18‐35 years who owned a smartphone and used LINE.	SG: 66 CG: 68 EG: 65 control: 47 (N=246 analyzed).	SG: 23.4 (4.2) CG: 25.2 (4.9) EG: 24.3 (4.3) control: 26.1 (4.9)	SG: 21/45 CG: 22/46 EG: 18/47 control: 15/32 (male/female)	Tracking-only control.
Alexandre et al 2025 [74]	France	2-arm parallel RCT CG: usual-care face-to-face supervised adapted physical activity program. IG: Kiplin digital gamified adapted physical activity program	Adults aged 18‐65 years treated for obesity or overweight or obesity with type 2 diabetes.	Randomized: IG: 25 CG: 25 Included in the modified intention-to-treat analysis: IG: 21 CG: 21	IG: 47.5 (11.0) CG: 47.89 (16.13)	IG: 17 female/4 male CG: 14 female/7 male	A 12-week hospital-based adapted physical activity program comprising 36 individual face-to-face supervised sessions (3 sessions/week). Sessions included warm-up, endurance and resistance exercises arranged as a 6-station circuit, and stretching; exercise intensity and resistance load were progressively increased.
Sañudo et al 2026 [77]	Spain	2-arm multicentre pilot RCT IG: IDHEApp gamified mHealth intervention plus Fitbit Charge 6 CG: usual routine with Fitbit Charge 6 used for measurement only	Young people with intellectual disabilities, including participants with Down syndrome and participants with mild-to-moderate intellectual disabilities.	Randomized IG: 30 CG: 30 Included in the complete-case analyses (38 participants total): IG: 23 CG: 15	Overall mean age: 20.4 years. Rome— IG: 24.0 (5.6) CG: 26.0 (5.3) Rijeka— IG: 17.0 (3.1) CG: 17.0 (3.3)	Overall: 41.2% male and 58.8% female	Participants continued their usual routines and wore a Fitbit Charge 6 solely for measurement.
Ming et al 2026 [78]	China	2-arm parallel group RCT IG: gamified team-based mHealth intervention CG: active technology-based PA monitoring	Full-time college students aged 18‐25 years who owned a smartphone and had BMI 18.5‐30.0.	Randomized: IG: 80 CG: 80 Completed T1: IG: 78 CG: 76 Included in the intention-to-treat analysis: IG: 80 CG: 80	IG: 20.5 (1.2) CG: 20.8 (1.3)	IG: 40 /40 CG: 40 /40	Participants used the same Shouti Fitness app, study-issued fitness watch, personalized daily and weekly PA goal prompts, and data-monitoring system as the IG.

a RCT: randomized controlled trials.

b IG: intervention group.

c CG: control group.

d Not available.

e MKMM: MobileKids Monster Manor.

f mHealth: mobile health.

g PA: physical activity.

h COOL: care & organize our lifestyle.

i TECH: technology-delivered physical activity intervention.

j VA: veterans affairs.

k T2M: time to move

l WHO: World Health Organization.

The characteristics of the study interventions are summarized in Table S2 (Multimedia Appendix 5). A majority of studies (38/53, 71.70%) incorporated a theoretical basis to guide the design of gamification interventions. These included behavioral economics principles (n=16), SDT (n=12), socio-cognitive theory (n=3), theory of planned behavior (n=3), health action process approach model (n=1), behavior change techniques (n=5), attribution theory (n=2), cognitive evaluation theory (n=1), transtheoretical model (n=2), self-regulation theory (n=1), fun theory (n=1), and social network theory (n=1), and the social identity approach (n=1). The duration of the interventions ranged from 1 to 48 weeks, with an average length of 14.4 weeks. Moreover, the types of systems used in these interventions varied, mainly consisting of software-based digital technologies (ie, applications, websites, and text messages) alongside hardware-based digital technologies (ie, wearable devices, accelerometers, and weight scales).

### Charted Outcome Domains and Reported Outcome Patterns

Table S2 (Multimedia Appendix 5) details the specific outcome changes observed in each included study, whereas Table 2 synthesizes the targeted outcome domains and the significance of intervention effects across studies. Collectively, these tables offer a comprehensive understanding of outcome selection and the effects of gamification interventions in the original studies. Among 53 studies included, outcomes were classified into 5 categories: PA-related outcomes, psychological outcomes, health outcomes, user retention outcomes, and knowledge-related cognitive outcomes. PA-related outcomes were reported 59 times, including step counts (n=34), minutes of moderate to vigorous physical activity (MVPA; n=13), minutes of walking (n=3), self-reported PA (n=5), PA intensity (n=1), PA frequency (n=1), and total amount of PA (n=2). Of these, 40 outcomes demonstrated statistically significant effects. Psychological outcomes are another area that has drawn researchers’ attention, particularly the constructs that support the theoretical foundations used in the studies. These outcomes included competency (n=3), PA self-efficacy (n=3), perceived enjoyment (n=2), autonomy (n=2), relatedness (n=2), PA motivation (n=2), as well as intention to engage in PA (n=1), life satisfaction (n=1), and mental well-being (n=1). Health outcomes were assessed in 8 of the included studies, with 5 showing significant results. User retention was examined in 4 studies, half of which reported statistically significant findings. Additionally, 2 studies whose interventions incorporated knowledge components also examined knowledge-related cognitive outcomes, with one reporting a significant effect.

**Table 2.** Categorization and statistical significance of diverse outcome domains (including physical activity, psychological, health, user retention, and cognitive metrics) reported across the 53 included randomized controlled trials.

Reported outcomes and studies	Significant effects^a^
PA^b^-related outcomes	
Step counts (n=34, 27√, 7×)	
Agarwal et al 2021 [26]	√
Alexander et al 2024 [31]	√
Francis et al 2021 [32]	√
Garde et al 2016 [34]	√
Garde et al 2018 [33]	√
Gremaud et al 2018 [37]	√
Greysen et al 2024 [39]	√
Hochsmann et al 2019 [42]	√
Lewey et al 2022 [46]	√
Mamede et al 2021 [49]	√
Nishiwaki et al 2014 [51]	√
Patel et al 2017 [54]	√
Patel et al 2019 [55]	√
Patel et al 2021 [53]	√
Patel et al 2021 [56]	√
Rungting Tu et al 2018 [65]	√
Santos et al 2021 [60]	√
Simmering et al 2025 [63]	√
Waddell et al 2022 [66]	√
Xu et al 2024 [67]	√
Gillcrist et al 2024 [35]	×
Gonze et al 2020 [36]	×
Greysen et al 2021 [38]	×
Lee et al 2022 [44]	×
Monroe et al 2023 [50]	×
Sanudo et al 2024 [61]	×
Maria do et al 2026 [75]	√
Patel et al 2025 [69]	√
Patel et al 2025 [70]	√
Janis et al 2025 [71]	×
Jeffrey Z et al 2026 [76]	√
Alexandre et al 2025 [74]	√
Sañudo et al 2026 [77]	√
Ming et al 2026 [78]	√
Minutes of MVPA^c^ (n=13, 5√, 8×)	
Greysen et al 2024 [39]	√
Maher et al 2015 [48]	√
Corepal et al 2019 [27]	×
Lee et al 2022 [44]	×
Leinonen et al 2017 [45]	×
Monroe et al 2023 [50]	×
Maria do et al 2026 [75]	×
Patel et al 2025 [69]	√
Janis et al 2025 [71]	×
Gaizka et al 2025 [73]	×
Alexandre et al 2025 [74]	√
Sañudo et al 2026 [77]	×
Ming et al 2026 [78]	√
Minutes of walking (n=3, 3√)	
Dadaczynski et al 2017 [28]	√
Maher et al 2015 [48]	√
Zuckerman et al 2014 [68]	√
Self-reported PA (n=5, 2√, 3×)	
Thorsteinsen et al 2014 [64]	√
Direito et al 2015 [29]	×
Mamede et al 2021 [49]	×
Janis et al 2025 [71]	×
Nimet et al 2025 [72]	√
PA intensity	
Lin et al 2021 [47]	×
PA frequency	
Nuijten et al 2022 [52]	×
Total amount of PA (n=2, 2√)	
Nishiwaki et al 2014 [51]	√
Gaizka et al 2025 [73]	√
Psychological outcomes	
Competency (n=3, 2√, 1×)	
Haque et al 2020 [40]	√
Hochsmann et al 2019 [41]	√
Direito et al 2015 [29]	×
PA self-efficacy (n=3, 1√)	
Dadaczynski et al 2017 [28]	√
Direito et al 2015 [29]	×
Robertson et al 2018 [58]	×
Perceived enjoyment (n=2, 1√, 1×)	
Hochsmann et al 2019 [41]	√
Direito et al 2015 [29]	×
Autonomy (n=2, 1√, 1×)	
Haque et al 2020 [40]	√
Direito et al 2015 [29]	×
Relatedness (n=2, 2×)	
Direito et al 2015 [29]	×
Haque et al 2020 [40]	×
PA motivation (n=2, 2√)	
Hochsmann et al 2019 [41]	√
Robertson et al 2020 [59]	√
Intention to engage in PA	
Dadaczynski et al 2017 [28]	√
Life satisfaction	
Pyky et al 2017 [57]	√
Mental well-being	
Corepal et al 2019 [27]	×
Health outcomes	
Knee Injury and Osteoarthritis Outcome Score	
Gillcrist et al 2024 [35]	√
Aerobic capacity	
Hochsmann et al 2019 [42]	√
Weight loss	
Kurtman et al 2018 [43]	√
Functional status and urgent care usage	
Greysen et al 2021 [38]	×
HbA_1c_^d^	
Hochsmann et al 2019 [42]	×
Sleep hours	
Sanudo et al 2024 [61]	×
Physical fitness (n=2, 2√)	
Gaizka et al 2025 [73]	√
Ming et al 2026 [78]	√
Body composition	
Ming et al 2026 [78]	√
Biometric outcomes	
Jeffrey Z et al 2026 [76]	×
User retention outcomes	
Game usage	
Hochsmann et al 2019 [41]	√
App usage	
Lee et al 2022 [44]	√
App attrition rate	
Edney et al 2019 [30]	×
Game engagement	
Schwarz et al 2021 [62]	×
Knowledge-related cognitive outcomes	
PA-related knowledge	
Dadaczynski et al 2017 [28]	√
Disease knowledge	
Lin et al 2021 [47]	×

a *P*<.05.

b PA: physical activity.

c MVPA: moderate to vigorous physical activity.

d HbA_1c_: hemoglobin A_1c_.

### Charted BCTs in Included Interventions

Among the 53 selected studies, 34 unique and independent interventions were included, with some interventions being repeated across different studies. Therefore, the BCT element analysis was conducted exclusively on these 34 distinct interventions. The most frequently used BCT categories were “2 feedback and monitoring” (34/34, 100%), “1 goals and planning” (31/34, 91.18%), and “6 comparison of behavior” (28/34, 82.35%), followed by “3 social support” (26/34, 76.47%), “10 reward and threat” (26/34, 76.47%), “12 antecedents” (17/34, 50.00%), “7 associations” (15/34, 44.12%), “4 shaping knowledge” (13/34, 38.24%), and “8 repetition and substitution” (9/34, 26.47%). The three least frequently used BCT categories were “5 natural consequences” (6/34, 17.65%), “9 comparison of outcomes” (3/34, 8.82%), and “11 regulation” (3/34, 8.82%).

The frequency of BCT element adoption is illustrated in Figure 2. Five BCTs were used in more than half of the included interventions: “2.2 feedback on behavior” (32/34, 94.12%), “1.1 goal setting (behavior)” (31/34, 91.18%), “6.2 social comparison” (27/34, 79.41%), “2.3 self-monitoring of behavior” (27/34, 79.41%), and “10.3 nonspecific reward” (23/34, 67.65%). Moreover, more than 30% of the specific interventions used the following 5 BCTs, “12.5 adding objects to the environment” (15/34, 44.12%), “7.1 prompts or cues” (15/34, 44.12%), “4.1 instruction on how to perform the behavior” (13/34, 38.24%), “3.1 social support (unspecified)” (12/34, 35.29%), and “3.3 social support (emotional)” (12/34, 35.29%).

**Figure 2.**
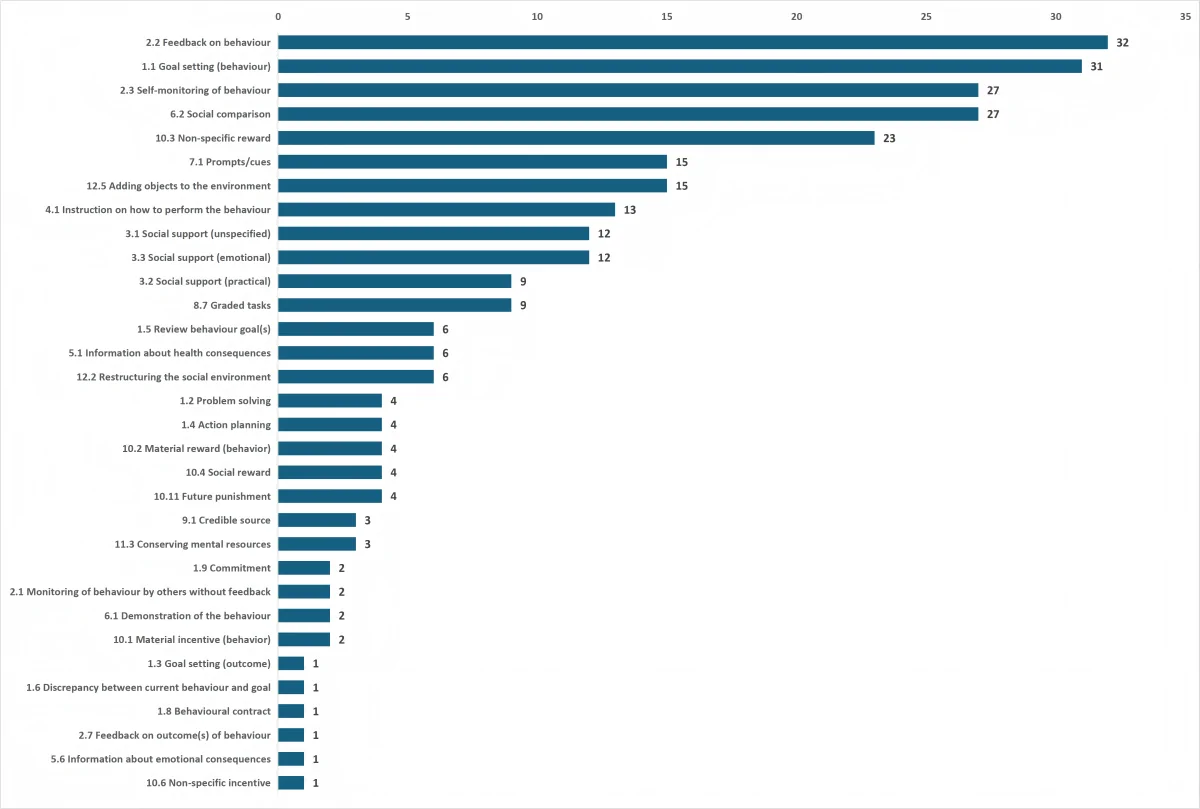
Frequency and distribution of specific behavior change techniques (BCTs) adopted across the 35 unique gamified physical activity interventions.

### Charted Gamification Elements and Octalysis Core Drives

#### Overview

Figure 3 illustrates the 48 gamification elements identified from the 34 independent interventions. Based on Chou’s Gamification Octalysis Framework, these elements are linked to 8 core drives (ie, epic meaning, accomplishment, empowerment, ownership, social influence, scarcity, unpredictability, and avoidance) that are designed to create engaging experiences and motivate user actions [16]. In our study, these drives are applied specifically to promote physical activity. We have categorized and summarized the gamification elements used in physical activity applications as expressions of these core drives. Multimedia Appendix 6 displays the gamification elements used in each individual intervention. The number of gamification elements across the included interventions varied from 3 to 13, with an average of 7.15. In total, the 34 interventions incorporated gamification elements 243 times.

**Figure 3.**
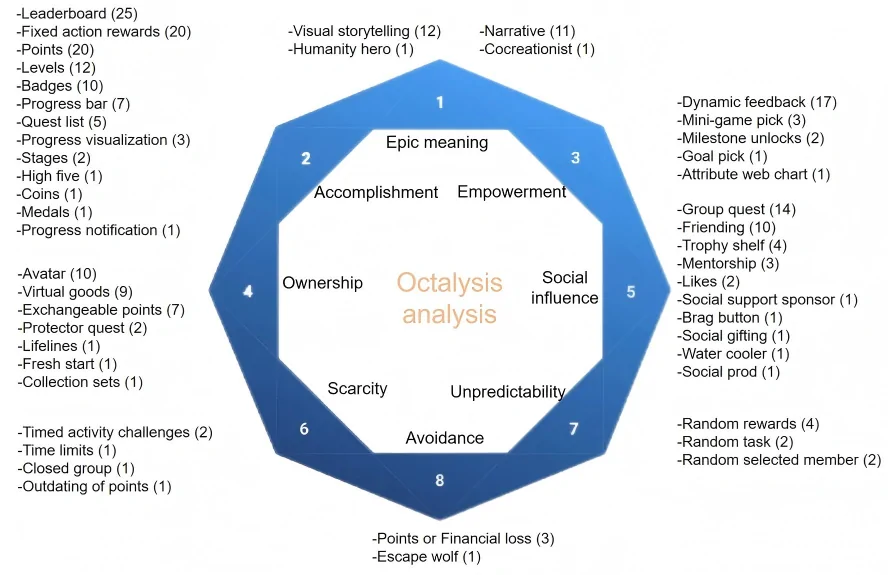
A comprehensive taxonomy of 48 gamification elements mapped to the 8 core psychological drives of the octalysis framework.

#### Gamification Elements Underlying the Eight Core Drives

Epic meaning refers to users participating in activities that are perceived as greater than themselves, and 44.12% of the included interventions incorporated this drive [16]. Four gamification elements were identified: visual storytelling (n=12), narrative (n=11), humanity hero (n=1), and cocreationist (n=1). The humanity hero element represents characters that embody noble ideas, and cocreationist refers to the collaborative creation of something. The second core drive is accomplishment (ie, making progress, developing skills, and eventually overcoming challenges), the most frequently used drive (32/34, 94.12%), including leaderboard (n=25), fixed action rewards (n=20)—rewards given after completing specific actions, points (n=20), levels (n=12), badges (n=10), progress bar (n=7), quest list (n=5), progress visualization (n=3)—any visual representation of progress, stages (n=2), high five (n=1)—a celebratory gesture, coins (n=1), medals (n=1), and progress notification (n=1). The next core drive is empowerment (21/34, 61.76%), which allows users to express their creativity. Five elements were associated with this drive. The most common outward manifestation was dynamic feedback (n=17). Other elements included choice options, such as mini-game pick (n=3), milestone unlocks (n=2), goal pick (n=1), while an attribute web chart (n=1) was used to visually display users’ capabilities.

Ownership (21/34, 61.76%) is the fourth core drive because users feel like they own something. Avatar (n=10), virtual goods (n=9), and exchangeable points (n=7) were the most frequently adopted gamification elements underlying ownership. Moreover, lifelines, collection sets, protector quest—tasks aimed at protecting something, and fresh start—an opportunity to begin something new or to restart were also included. Social influence (20/34, 58.82%), the fifth core drive, included elements such as group quest (n=14), friending (n=10), trophy shelf (n=4)—a space for displaying trophies, badges, medals, etc, mentorship (n=3), likes (n=2), social support sponsor (n=1), brag button (n=1)—a feature that allows individuals to publicly share or view achievements, social gifting (n=1)—giving virtual gifts to others to enhance social connections, water cooler (n=1)—a place for casual chatting among users, and social prod (n=1)—strategies or techniques used to encourage social behaviors.

The remaining three core drives (ie, scarcity, unpredictability, avoidance) are classified as black hat core drives by Chou [16]. These drives are often controversial because they make users unsure of what will happen next and afraid of losing something. Therefore, they are less commonly used in the interventions included in this study. In the scarcity drive (5/34, 14.71%), motivation was created through elements such as timed activity challenges (n=2), time limits (n=1), closed group (n=1)—where not everyone is allowed to join, and outdating of points (n=1). Unpredictability (8/34, 23.53%) involves uncertain outcomes, including random rewards (n=4), random task (n=2), and random selected member (n=2)—who determines the group’s future. Avoidance (4/34, 11.76%) was represented by points or financial loss (n=3), and escape wolf (n=1)— which uses time pressure to drive users to act quickly.

### BCT—Gamification Element Mappings

The BCT—gamification element mapping showed that several BCTs were conceptually aligned with gamification elements through shared motivational targets. The detailed mapping results are shown in Figure 4. The majority of BCTs were mapped to empowerment (n=10) and accomplishment (n=5), with no BCTs classified under the core motivations of scarcity or unpredictability. Feedback on or outcomes of behavior and social support were represented by the widest range of gamification elements, with 8 and 5 elements mapped to these BCTs, respectively. Some BCTs (ie, information about consequences, review behavior goals, conserving mental resources, credible source, action planning, demonstration of the behavior, behavioral contract, and problem solving) were not explicitly represented by gamification elements in the included interventions, suggesting potential gaps in the integration of reflective, planning-oriented, and instructional BCTs into gamified PA intervention designs.

**Figure 4.**
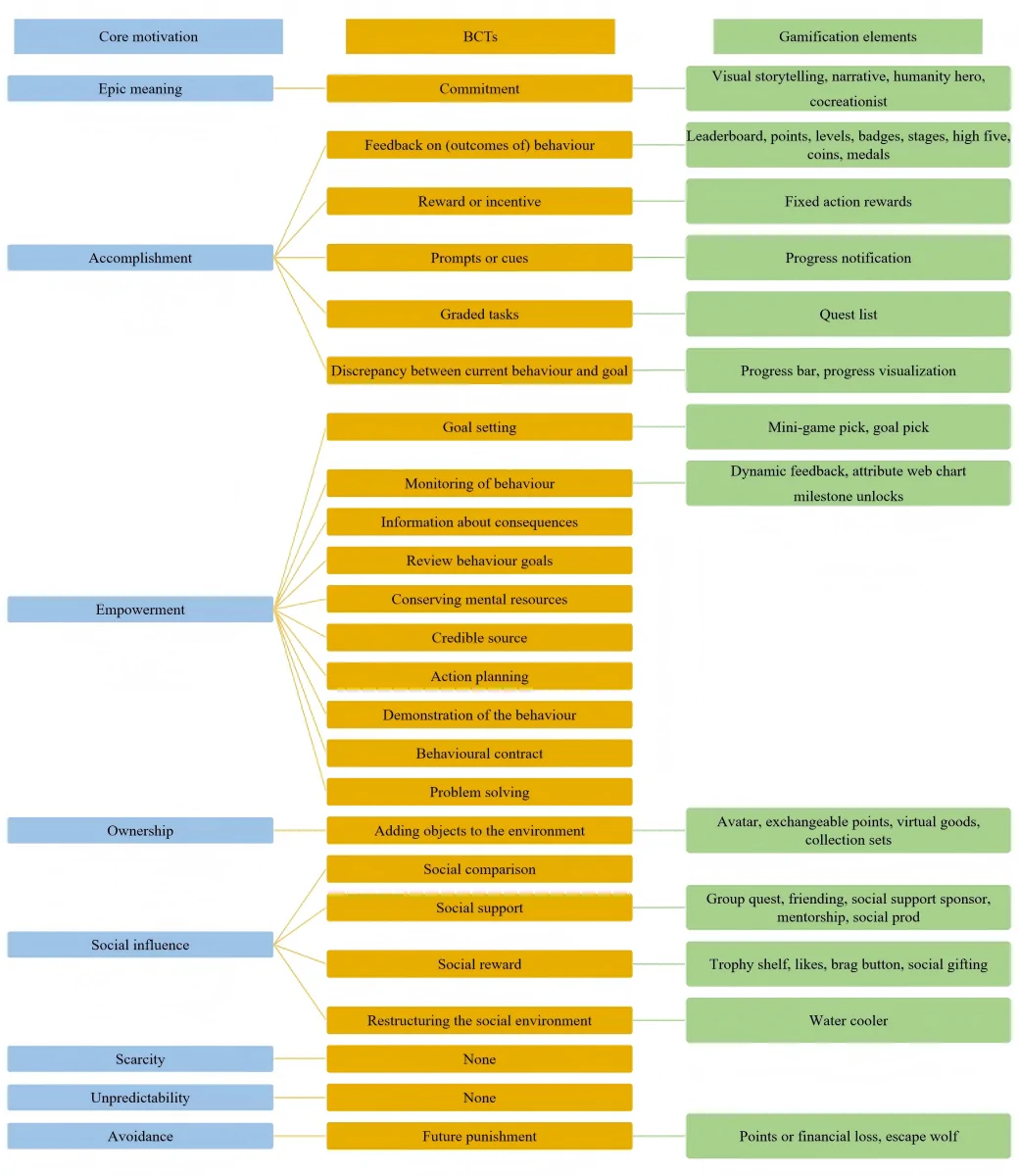
Cross-mapping analysis illustrating the conceptual alignment and integration gaps between applied behavior change techniques (BCTs) and octalysis-based gamification. BCT: behavior change technique.

## Discussion

### Principal Findings

The scoping review mapped the evidence on gamification elements used in PA interventions, categorized these elements according to the Octalysis Framework, and identified their links with BCTs. Following a comprehensive search across seven databases, 53 RCTs and 34 unique interventions were included in this review. The majority of studies were conducted in developed regions, such as the USA and European countries. These studies targeted diverse populations, including individuals both with and without medical conditions, spanning age groups from children to older adults. Behavioral economics principles emerged as the most commonly applied theoretical framework. Most interventions were integrated with digital hardware technologies, such as wearable devices. The studies reported a variety of outcomes, including user retention, behavioral changes, psychological impacts, and health-related indicators. The most frequently adopted BCT elements were “2.2 feedback on behavior” and “1.1 goal setting (behavior).” Moreover, using the Octalysis framework, 48 gamification elements across 8 core motivational drives were identified among the 34 interventions. These findings offer meaningful insights for informing the design of future gamification strategies aimed at promoting PA.

The main contribution of this review is not to estimate the overall effectiveness of gamified PA interventions, but to show how these interventions are designed. Specifically, this review provides 3 main findings: a summary of existing gamified PA trials, a classification of the gamification elements used in these interventions based on the Octalysis framework, and a comparison between these gamification elements and BCTs. Together, these findings show how game design is used to support motivation in current interventions and highlight areas where intervention design could be improved. The resulting taxonomy and mapping may serve as a design-oriented resource for researchers and digital health developers by supporting the more deliberate selection, combination, and reporting of gamification elements according to the intended motivational and behavioral objectives of future PA interventions.

### Interpretation of Study and Intervention Characteristics

Gamification interventions in PA promotion have rapidly emerged and developed over the past decade, with the majority of studies conducted in developed countries. However, the reach of these interventions may be limited in developing and underdeveloped regions, where access to technology and health care resources is often restricted. This raises concerns about health equity, as disadvantaged populations may be left out of the potential benefits of gamified PA programs [79]. Future research should focus on addressing these barriers, considering the technological and socio-economic challenges faced by underserved areas.

Behavioral economics principles were the most frequently used theoretical framework in the 53 studies, with 13 of these studies conducted by a team from the University of Pennsylvania, USA, who applied these principles to guide their interventions. Although widely accepted and used in original studies, the long-term sustainability of behavioral economics-informed interventions to promote PA remains uncertain [80,81]. This may be due to the fact that behavioral economics principles primarily rely on strategies and techniques such as loss aversion, prospect theory, and nudges, which provide short-term incentives to influence immediate health behavior decisions [82]. However, these interventions are often driven by short-term interests, and without continued incentives, the health behavior changes are unlikely to be sustained [83]. Another commonly used theory was SDT, which highlights the crucial role of motivation in promoting behaviors. Compared to other theoretical frameworks, it has the potential to foster long-term health behaviors if individuals’ autonomous or intrinsic motivation is effectively stimulated [8]. Therefore, different theories serve distinct purposes, reflecting different motivational pathways and intervention design priorities. Future research should examine how different theoretical traditions are represented and operationalized in gamified PA interventions and how they may support different motivational mechanisms. Intervention duration is a crucial aspect of intervention design, as the timeframe plays a key role in establishing sustainable health behaviors such as PA. In the included studies, the average intervention period was 14.4 weeks, falling short of the recommended 24-week duration needed for PA maintenance [84,85]. Therefore, more long-term intervention studies are needed in the future to better understand longitudinal changes in PA behavior and to refine intervention design, reporting, and long-term evaluation.

### Mapping of Reported Outcome Domains

The outcomes examined in the 53 included studies varied, covering aspects such as user retention, psychological responses, behavioral changes, and health-related indicators, reflecting the full pathway of health behavior change. Gamification, as a persuasive technology, plays a critical role in supporting this process by enhancing user retention [86]. Continued engagement with gamified elements may influence users’ psychological states, which can subsequently drive behavioral changes like increased PA, and may contribute to improved health-related outcomes. The included studies reported heterogeneous outcome patterns, with light PA, such as step counts and minutes of walking, showing the most significant improvements. To achieve greater health benefits, future research should focus more on moderate to vigorous PA, as well as the psychological responses that underpin these behaviors.

### Interpretation of BCT Mapping and Integration Gaps

The three most commonly used BCTs were “2.2 feedback on behavior,” “1.1 goal setting (behavior),” and “6.2 social comparison.” These techniques are popular due to their broad acceptance and ease of implementation. However, this distribution indicates that current gamified PA interventions rely heavily on a small group of BCTs. Given the complexity of behavioral interventions, assessing the effectiveness of any single BCT is challenging. Rather than focusing on the quantity of BCTs used, the quality of their application is of greater importance [87,88]. Taking “2.2 feedback on behavior” as an example, feedback can vary in terms of being generalized or personalized, static or dynamic, and visualized or nonvisualized, all of which significantly impact user experiences [89]. Therefore, the integration of BCTs with design strategies is crucial, yet also challenging. The mapping also revealed several BCTs that were weakly represented or not clearly embodied by gamification elements, such as action planning, problem solving, credible source, and information about consequences. These gaps suggest that future gamified PA interventions should more deliberately align game mechanics with behavior change functions, rather than simply adding more game elements.

### Interpretation of Octalysis Taxonomy and Design Implications

To the best of our knowledge, this is the first review to comprehensively summarize the use of gamification elements in existing published PA promotion applications, providing a valuable reference for the design of future gamified PA interventions. The core contribution and essence of the Gamification Octalysis Framework lies in its ability to connect gamification elements with individual motivation [16]. From the perspective of human-focused design, it emphasizes that personal motivation takes precedence over functionality [16]. The framework identifies 8 core motivational drives. Our findings show that the accomplishment drive was the most commonly used, as the gamified elements associated with this drive were easy to implement. Traditional gamified elements, such as points, badges, and leaderboards (PBL), were categorized under this drive. However, the presence of a gamification element alone does not necessarily indicate that its intended motivational mechanism has been achieved. Another significant contribution of the Gamification Octalysis Framework is its classification of core drives into intrinsic motivation (ie, empowerment, social influence, and unpredictability) and extrinsic motivation (ie, accomplishment, ownership, and scarcity) [16]. The ultimate goal of PA stimulation may lie in the combined use of both extrinsic and intrinsic gamified elements, with the transformation of extrinsic motivation into intrinsic motivation based on SDT [8].

Beyond the use of gamified elements, the underlying rules and mechanisms of these games may play an even more crucial role. The design of gamification presents a significant challenge in current PA promotion applications. Involving users in the design of gamification elements could offer a potential solution to this problem [90,91]. Other approaches, such as personalization, should also be taken into account when designing future gamification interventions [92,93]. Taken together, these findings suggest that future gamified PA interventions should be designed by first identifying the intended motivational pathway, rather than by simply adding common game elements. For example, if the goal is to support competence and progress, accomplishment-based elements such as goals, levels, progress bars, and feedback may be appropriate; if the goal is to strengthen social connectedness, social influence elements such as group quests, peer support, and shared challenges may be more suitable. The mapping between gamification elements and BCTs also suggests that future interventions should give more attention to underrepresented behavior change functions, such as action planning, problem solving, and information about consequences. Therefore, the design value of this review lies in helping researchers select game elements more deliberately and align them with both motivational drives and behavior change functions.

### Limitations

Firstly, we exclusively included RCTs in this review, while we acknowledge that other types of study designs, such as quasi-experimental studies, have also investigated gamified PA application interventions, our focus on RCTs was intentional. We limited the sources of evidence to RCTs because our objective was to map gamification elements and BCTs in interventions that had been formally tested. However, this decision may have excluded formative studies, feasibility studies, qualitative evaluations, and design papers that could contain innovative or emerging gamification elements. Secondly, we identified BCTs and summarized the gamification elements present in the 34 unique interventions included in our review. Although the coding was conducted independently by 2 researchers (SJL and LQ) and discrepancies were resolved through discussion, the process is inherently subject to some degree of subjective interpretation. This subjectivity may have influenced the final coding outcomes. Thirdly, due to the wide range of outcomes assessed across the original RCTs and the limited number of studies reporting each specific outcome, we were unable to perform a meta-analysis to quantitatively estimate pooled effects. Instead, we provided a descriptive summary of reported outcome patterns. Nevertheless, the primary focus of this review was the systematic identification and analysis of gamification elements, guided by the Gamification Octalysis Framework. Lastly, although the eligibility criteria allowed studies involving any human population, the identified evidence was predominantly based on adults, with only a small number of studies involving children or adolescents. Therefore, the findings of this review should be interpreted mainly in relation to adult populations and generalized to children and adolescents with caution.

### Conclusions

This scoping review complements previous effectiveness-focused reviews by examining how gamified PA interventions are designed and which motivational and behavior change functions they target. By developing an Octalysis-based taxonomy of 48 gamification elements and mapping these elements against BCTs, this review provides a descriptive, design-oriented evidence map of commonly used design patterns and underrepresented behavior change functions. The findings may support more deliberate and transparent selection, combination, and reporting of gamification elements in future PA interventions. However, they should not be interpreted as evidence of the effectiveness of individual gamification elements, and further research is needed to evaluate how specific design configurations influence engagement and PA outcomes.

## Supplementary material

10.2196/85804 Multimedia Appendix 1 Search strategies used for the literature search.

10.2196/85804 Multimedia Appendix 2 BCT (v1): 93 hierarchically clustered techniques.

10.2196/85804 Multimedia Appendix 3 Figure S1 Octalysis framework by Chou.

10.2196/85804 Multimedia Appendix 4 Exclusion reasons.

10.2196/85804 Multimedia Appendix 5 Intervention components, theoretical foundations, delivery systems, intervention duration (time frame), and effects on primary and secondary outcomes for the 53 included randomized controlled trials.

10.2196/85804 Multimedia Appendix 6 Gamification elements in the included interventions (Figure S2 to improve clarity).

10.2196/85804 Checklist 1 PRISMA-S Checklist 1

10.2196/85804 Checklist 2 PRISMA-SCR Checklist 2

## Abbreviations

BCT: behavior change technique
BCTT: BCT Taxonomy
EBSCOhost: Elton B. Stephens Company host
MVPA: moderate to vigorous physical activity
PA: physical activity
PBL: points, badges, and leaderboards
PCC: population, concept, context
PRISMA-S: Preferred Reporting Items for Systematic reviews and Meta-Analyses literature search extension
PRISMA-ScR: Preferred Reporting Items for Systematic Reviews and Meta-Analyses extension for Scoping Reviews
PROSPERO: International Prospective Register of Systematic Reviews
RCT: randomized controlled trial
SDT: self-determination theory
WHO: World Health Organization
