# Supplementary material for: Gamification Elements in Physical Activity Interventions: Scoping Review and Taxonomy Based on the Octalysis Framework
Source: JMIR Serious Games. 2026 Sep 25;14:e85804. doi: 10.2196/85804 (PMC13618205; doi:10.2196/85804)
Supplement: Supplementary file 1 [file games-v14-e85804-s002.pdf]

Table S2

BCT Taxonomy (v1): 93 hierarchically-clustered techniques

Page	Grouping and BCTs
1	**1. Goals and planning**
	1.1. Goal setting (behavior)<br>1.2. Problem solving<br>1.3. Goal setting (outcome)<br>1.4. Action planning<br>1.5. Review behavior goal(s)<br>1.6. Discrepancy between current behavior and goal<br>1.7. Review outcome goal(s)<br>1.8. Behavioral contract<br>1.9. Commitment
3	**2. Feedback and monitoring**
	2.1. Monitoring of behavior by others without feedback<br>2.2. Feedback on behaviour<br>2.3. Self-monitoring of behaviour<br>2.4. Self-monitoring of outcome(s) of behaviour<br>2.5. Monitoring of outcome(s) of behavior without feedback<br>2.6. Biofeedback<br>2.7. Feedback on outcome(s) of behavior
5	**3. Social support**
	3.1. Social support (unspecified)<br>3.2. Social support (practical)<br>3.3. Social support (emotional)
6	**4. Shaping knowledge**
	4.1. Instruction on how to perform the behavior<br>4.2. Information about Antecedents<br>4.3. Re-attribution<br>4.4. Behavioral experiments
7	**5. Natural consequences**
	5.1. Information about health consequences<br>5.2. Salience of consequences<br>5.3. Information about social and environmental consequences<br>5.4. Monitoring of emotional consequences<br>5.5. Anticipated regret<br>5.6. Information about emotional consequences
8	**6. Comparison of behaviour**
	6.1. Demonstration of the behavior<br>6.2. Social comparison<br>6.3. Information about others' approval
9	**7. Associations**
	7.1. Prompts/cues<br>7.2. Cue signalling reward<br>7.3. Reduce prompts/cues<br>7.4. Remove access to the reward<br>7.5. Remove aversive stimulus<br>7.6. Satiation<br>7.7. Exposure<br>7.8. Associative learning
10	**8. Repetition and substitution**
	8.1. Behavioral practice/rehearsal<br>8.2. Behavior substitution<br>8.3. Habit formation<br>8.4. Habit reversal<br>8.5. Overcorrection<br>8.6. Generalisation of target behavior<br>8.7. Graded tasks
11	**9. Comparison of outcomes**
	9.1. Credible source<br>9.2. Pros and cons<br>9.3. Comparative imagining of future outcomes
12	**10. Reward and threat**
	10.1. Material incentive (behavior)<br>10.2. Material reward (behavior)<br>10.3. Non-specific reward<br>10.4. Social reward<br>10.5. Social incentive<br>10.6. Non-specific incentive<br>10.7. Self-incentive<br>10.8. Incentive (outcome)<br>10.9. Self-reward<br>10.10. Reward (outcome)<br>10.11. Future punishment
15	**11. Regulation**
	11.1. Pharmacological support<br>11.2. Reduce negative emotions<br>11.3. Conserving mental resources<br>11.4. Paradoxical instructions
16	**12. Antecedents**
	12.1. Restructuring the physical environment<br>12.2. Restructuring the social environment<br>12.3. Avoidance/reducing exposure to cues for the behavior<br>12.4. Distraction<br>12.5. Adding objects to the environment<br>12.6. Body changes
17	**13. Identity**
	13.1. Identification of self as role model<br>13.2. Framing/reframing<br>13.3. Incompatible beliefs<br>13.4. Valued self-identify<br>13.5. Identity associated with changed behavior
18	**14. Scheduled consequences**
	14.1. Behavior cost<br>14.2. Punishment<br>14.3. Remove reward<br>14.4. Reward approximation<br>14.5. Rewarding completion<br>14.6. Situation-specific reward<br>14.7. Reward incompatible behavior<br>14.8. Reward alternative behavior<br>14.9. Reduce reward frequency<br>14.10. Remove punishment
19	**15. Self-belief**
	15.1. Verbal persuasion about capability<br>15.2. Mental rehearsal of successful performance<br>15.3. Focus on past success<br>15.4. Self-talk
19	**16. Covert learning**
	16.1. Imaginary punishment<br>16.2. Imaginary reward<br>16.3. Vicarious consequences
