# Supplementary material for: Gamification Elements in Physical Activity Interventions: Scoping Review and Taxonomy Based on the Octalysis Framework
Source: JMIR Serious Games. 2026 Sep 25;14:e85804. doi: 10.2196/85804 (PMC13618205; doi:10.2196/85804)
Supplement: Supplementary file 2 [file games-v14-e85804-s001.docx]

| Table S1 Search strategy  From inception to 2026/07/07 | |
| --- | --- |
| PubMed Platform: PubMed | |
| #1 | "gamification"[MeSH Terms] OR "gamified"[Title/Abstract] OR "game"[Title/Abstract] OR "gaming"[Title/Abstract] OR "gameful"[Title/Abstract] OR "game mechani*"[Title/Abstract] OR "game element*"[Title/Abstract] OR "game feature*"[Title/Abstract] OR "game design*"[Title/Abstract] OR "gamifying"[Title/Abstract] |
| #2 | "exercise"[MeSH Terms] OR "exercis*"[Title/Abstract] OR "physical activit*"[Title/Abstract] OR "physical exercis*"[Title/Abstract] OR "physical fitness"[Title/Abstract] OR "sedentary"[Title/Abstract] OR "inactivity"[Title/Abstract] OR "sport*"[Title/Abstract] |
| #3 | "randomized controlled trial"[Publication Type] OR "controlled clinical trial"[Publication Type] OR "randomized"[Title/Abstract] OR "placebo"[Title/Abstract] OR "randomly"[Title/Abstract] OR "randomised"[Title/Abstract] |
| #4 | #1 AND #2 AND #3 |
| Embase Platform: Elsevier | |
| #1 | gamification:ti,ab,kw OR gamified:ti,ab,kw OR game:ti,ab,kw OR gaming:ti,ab,kw OR gameful:ti,ab,kw OR 'game mechani*':ti,ab,kw OR 'game element*':ti,ab,kw OR 'game feature*':ti,ab,kw OR 'game design*':ti,ab,kw OR 'gamifying':ti,ab,kw |
| #2 | 'gamification'/exp |
| #3 | #1 OR #2 |
| #4 | exercise:ti,ab,kw OR exercis*:ti,ab,kw OR 'physical activit':ti,ab,kw OR 'physical exercis*':ti,ab,kw OR 'physical fitness':ti,ab,kw OR sedentary:ti,ab,kw OR inactivity:ti,ab,kw OR sport*:ti,ab,kw |
| #5 | 'exercise'/exp |
| #6 | #4 OR #5 |
| #7 | 'randomized controlled trial':it OR 'controlled clinical trial':it OR randomized:ti,ab,kw OR placebo:ti,ab,kw OR randomly:ti,ab,kw OR randomised:ti,ab,kw |
| #8 | #3 AND #6 AND #7 |
| Cochrane library Platform: Wiley Online Library | |
| #1 | (Gamification):ab,ti,kw OR (Gamified):ab,ti,kw OR (Game):ab,ti,kw OR (Gaming):ab,ti,kw OR (Gameful):ab,ti,kw OR (Game mechani*):ab,ti,kw OR (Game element*):ab,ti,kw OR (Game feature*):ab,ti,kw OR (Game design*):ab,ti,kw OR (Gamifying):ab,ti,kw |
| #2 | MeSH descriptor: [Gamification] explode all trees |
| #3 | #1 OR #2 |
| #4 | (Exercise):ab,ti,kw OR (exercis*):ab,ti,kw OR (physical activit):ab,ti,kw OR (physical exercis*):ab,ti,kw OR (physical fitness):ab,ti,kw OR (sedentary):ab,ti,kw OR (inactivity):ab,ti,kw OR (sport*):ab,ti,kw |
| #5 | MeSH descriptor: [Exercise] explode all trees |
| #6 | #4 OR #5 |
| #7 | (randomized controlled trial):pt OR (controlled clinical trial):pt OR (randomized):ti,ab,kw OR (placebo):ti,ab,kw OR (randomly):ti,ab,kw OR (randomised):ti,ab,kw |
| #8 | #3 AND #6 AND #7 |
| Web of Science Platform: Clarivate Analytics | |
| #1 | TS= (Gamification OR Gamified OR Game OR Gaming OR Gameful OR Game mechani* OR Game element* OR Game feature* OR Game design* OR Gamifying) |
| #2 | TS= (Exercise OR exercis* OR physical activit OR physical exercis* OR physical fitness OR sedentary OR inactivity OR sport*) |
| #3 | TS= (Randomized controlled trial OR controlled clinical trial OR Randomized OR Placebo OR Randomly OR Randomised) |
| #4 | #1 AND #2 AND #3 |
| CINAHL Platform: EBSCOhost | |
| #1 | TI (Gamification OR Gamified OR Game OR Gaming OR Gameful OR Game mechani* OR Game element* OR Game feature* OR Game design* OR Gamifying) OR AB (Gamification OR Gamified OR Game OR Gaming OR Gameful OR Game mechani* OR Game element* OR Game feature* OR Game design*OR Gamifying) |
| #2 | TI (Exercise OR exercis* OR physical activit OR physical exercis* OR physical fitness OR sedentary OR inactivity OR sport*) OR AB (Exercise OR exercis* OR physical activit OR physical exercis* OR physical fitness OR sedentary OR inactivity OR sport*) |
| #3 | TI (Randomized controlled trial OR controlled clinical trial OR Randomized OR Placebo OR Randomly OR Randomised) OR AB (Randomized controlled trial OR controlled clinical trial OR Randomized OR Placebo OR Randomly OR Randomised) |
| #4 | #1 AND #2 AND #3 |
| SPORTDiscus Platform: EBSCOhost | |
| #1 | TI (Gamification OR Gamified OR Game OR Gaming OR Gameful OR Game mechani* OR Game element* OR Game feature* OR Game design* OR Gamifying) OR AB (Gamification OR Gamified OR Game OR Gaming OR Gameful OR Game mechani* OR Game element* OR Game feature* OR Game design*OR Gamifying) |
| #2 | TI (Exercise OR exercis* OR physical activit OR physical exercis* OR physical fitness OR sedentary OR inactivity OR sport*) OR AB (Exercise OR exercis* OR physical activit OR physical exercis* OR physical fitness OR sedentary OR inactivity OR sport*) |
| #3 | TI (Randomized controlled trial OR controlled clinical trial OR Randomized OR Placebo OR Randomly OR Randomised) OR AB (Randomized controlled trial OR controlled clinical trial OR Randomized OR Placebo OR Randomly OR Randomised) |
| #4 | #1 AND #2 AND #3 |
| PsycINFO Platform: EBSCOhost | |
| #1 | TI (Gamification OR Gamified OR Game OR Gaming OR Gameful OR Game mechani* OR Game element* OR Game feature* OR Game design* OR Gamifying) OR AB (Gamification OR Gamified OR Game OR Gaming OR Gameful OR Game mechani* OR Game element* OR Game feature* OR Game design*OR Gamifying) |
| #2 | TI (Exercise OR exercis* OR physical activit OR physical exercis* OR physical fitness OR sedentary OR inactivity OR sport*) OR AB (Exercise OR exercis* OR physical activit OR physical exercis* OR physical fitness OR sedentary OR inactivity OR sport*) |
| #3 | TI (Randomized controlled trial OR controlled clinical trial OR Randomized OR Placebo OR Randomly OR Randomised) OR AB (Randomized controlled trial OR controlled clinical trial OR Randomized OR Placebo OR Randomly OR Randomised) |
| #4 | #1 AND #2 AND #3 |
