# Supplementary material for: Gamification Elements in Physical Activity Interventions: Scoping Review and Taxonomy Based on the Octalysis Framework
Source: JMIR Serious Games. 2026 Sep 25;14:e85804. doi: 10.2196/85804 (PMC13618205; doi:10.2196/85804)
Supplement: Supplementary file 3 [file games-v14-e85804-s004.docx]

| Table S3 Studies excluded at full-text review | | |
| --- | --- | --- |
| Number | Reference | Reason |
| 1 | Online platform for healthy weight loss in adults with overweight and obesity - the "POEmaS" project: a randomized controlled trial. | Wrong study design |
| 2 | An online game-based intervention using quizzes to improve nutrition and physical activity outcomes among university students. | Wrong intervention |
| 3 | Efficacy of a Mobile Health App (eMOTIVA) Regarding Compliance With Cardiac Rehabilitation Guidelines in Patients With Coronary Artery Disease: Randomized Controlled Clinical Trial. | Wrong intervention |
| 4 | Individual differences in regulatory mode moderate the effectiveness of a pilot mHealth trial for diabetes management among older veterans. | Wrong outcome |
| 5 | Usage and Daily Attrition of a Smartphone-Based Health Behavior Intervention: Randomized Controlled Trial. | Wrong intervention |
| 6 | HelperFriend, a Serious Game for Promoting Healthy Lifestyle Behaviors in Children: Design and Pilot Study. | Wrong intervention |
| 7 | The effect of a game-based mobile app 'MyHeartMate' to promote lifestyle change in coronary disease patients: a randomized controlled trial. | Wrong intervention |
| 8 | A Remote Behaviorally Designed Intervention to Promote Physical Activity in Patients With Knee Osteoarthritis: Results of a Pilot Randomized Clinical Trial. | Wrong outcome |
| 9 | Gamification as a Tool for Promoting Physical Exercise and Healthy Eating Habits in Healthcare Worker Women: Effects on Cardiometabolic Health and Physical Fitness at Workplace. | Wrong intervention |
| 10 | Wellness partners: design and evaluation of a web-based physical activity diary with social gaming features for adults. | Wrong intervention |
| 11 | Push Notifications From a Mobile App to Improve the Body Composition of Overweight or Obese Women: Randomized Controlled Trial. | Wrong outcome |
| 12 | Improving adherence and outcomes in diabetic patients. | Wrong intervention |
| 13 | Effects of a Mobile App to Promote Social Participation on Older Adults: Randomized Controlled Trial. | Wrong intervention |
| 14 | A Remote Behaviorally-Designed Intervention to Promote Physical Activity in Patients with Knee Osteoarthritis: Results of a Pilot Randomized Clinical Trial. | Duplicate |
| 15 | Effectiveness of a nurse-led mHealth approach in preventing obesity in adolescents: an intervention study. | Wrong intervention |
| 16 | Effectiveness of a Gamification Strategy to Prevent Childhood Obesity in Schools: A Cluster Controlled Trial. | Wrong intervention |
| 17 | Feasibility of a Sensor-Controlled Digital Game for Heart Failure Self-management: Randomized Controlled Trial. | Wrong intervention |
| 18 | An early phase trial testing the proof of concept for a gamified smartphone app in manipulating automatic evaluations of exercise. | Wrong outcome |
| 19 | Efficacy of a Mobile Health-Based Behavioral Treatment for Lifestyle Modification in Type 2 Diabetes Self-Management: Greenhabit Randomized Controlled Trial. | Wrong intervention |
| 20 | Effect of a multi-disciplinary program on anthropometric and biochemical parameters in obese and overweight elementary school girls: A randomized clinical trial. | Wrong intervention |
| 21 | Stepping into survivorship pilot study: Harnessing mobile health and principles of behavioral economics to increase physical activity in ovarian cancer survivors. | Wrong study design |
| 22 | Evaluation of a Serious Self-Regulation Game Intervention for Overweight-Related Behaviors ("Balance It"): A Pilot Study. | Wrong intervention |
| 23 | Efficacy of the Aim2Be Intervention in Changing Lifestyle Behaviors Among Adolescents With Overweight and Obesity: Randomized Controlled Trial. | Wrong intervention |
| 24 | Effectiveness of home-based and remotely supervised aerobic exercise in Parkinson's disease: a double-blind, randomised controlled trial. | Wrong outcome |
| 25 | A Serious Game for the Prevention of Obesity in School Children-Impact of Parent's Involvement: A Randomized Controlled Trial. | Wrong intervention |
| 26 | Stepping into survivorship: harnessing mobile health and principles of behavioral economics to increase physical activity in ovarian cancer survivors. | Duplicate |
| 27 | Everyday Digital Support to Promote Health and Literacy Among Older Adults: 14-Week Randomized Digital Pilot Trial by Engagement Level. | Wrong intervention |
| 28 | Gamified Learning in Higher Vocational Physical Education: A Four-Group Randomized Controlled Trial. | Wrong intervention |
| 29 | Evaluating the usability of Diactive-1: mHealth for personalized exercise and education in children and adolescents with type 1 diabetes. | Wrong outcome |
| 30 | Digital motivation: fitness apps and student physical activity. | Wrong intervention |
| 31 | Effectiveness of remote gamification pulmonary rehabilitation intervention based on the health action process approach theory in older adults with chronic obstructive pulmonary disease: a pilot randomized controlled trial. | Wrong intervention |
| 32 | Effectiveness of Mobile Health Applications for Cardiometabolic Risk Reduction in Urban and Rural India: A Pilot, Randomized Controlled Study. | Wrong intervention |
| 33 | Efficacy of a Mobile Health–Based Behavioral Treatment for Lifestyle Modification in Type 2 Diabetes Self-Management: Greenhabit Randomized Controlled Trial. | Wrong intervention |
| 34 | Autonomous Motivation as a Mechanism of Change in a Gamified Digital Physical Activity Intervention: A Randomized Controlled Trial. | Full text unavailable |
| 35 | The mLIFE randomized trial examining the impact of gamifying social support provision for weight loss. | Wrong intervention |
