# Supplementary material for: Gamification Elements in Physical Activity Interventions: Scoping Review and Taxonomy Based on the Octalysis Framework
Source: JMIR Serious Games. 2026 Sep 25;14:e85804. doi: 10.2196/85804 (PMC13618205; doi:10.2196/85804)
Supplement: Supplementary file 4 [file games-v14-e85804-s005.docx]

| Table S4. Intervention components, theoretical foundations, delivery systems, intervention duration (time frame), and effects on primary and secondary outcomes for the 53 included randomized controlled trials. Abbreviation: PA, physical activity; RCT, randomized controlled trials; IG, intervention group; CG, control group; MVPA, moderate to vigorous physical activity; SDT, self-determination theory; PCP, primary care physician; ASD, autism spectrum disorder; SE, standard error; COOL, care & organize our lifestyle; BCT, behavior change technique; MD, mean difference. | | | | | | | | | | | | | | | | | |
| --- | --- | --- | --- | --- | --- | --- | --- | --- | --- | --- | --- | --- | --- | --- | --- | --- | --- |
| Author | | Study arms | | Intervention contents | | Theoretical basis | | Duration (weeks) | | Type of systems | | Primary outcome | | Secondary outcome | | Conclusion | |
| Agarwal | | 3-arm RCT; IG1: gamification with social support, IG2: gamification with social support and loss-framed financial incentives, CG: feedback only. | | All participants received a wearable device to track step counts and selected a step goal. Participants in the 2 gamification groups were entered into a 12-week game. | | behavioral economics principles | | 12 | | Application and wearable device. | | Mean daily steps by wearable device:  IG1-CG: Increased 433 steps (*P* = .81);  IG2-CG: Increased 1224 steps (*P* = .005). | | Proportion of days meeting their step goal:  IG1-CG: Increased 0.21 participant-day (*P* < .001);  IG2-CG: Increased 0.34 participant-day (*P* < .001). | | Effective for promoting physical activity in IG2 but not in IG1. | |
| Corepal | | 2-arm RCT; IG: The StepSmart Challenge, CG: no intervention control group. | | The StepSmart Challenge used gamification strategies, combined with the core tenets of self-determination theory, to encourage and support physical activity behavior change by moving participants along the motivation continuum from more external forms of motivation towards intrinsic motivation. | | Self-determination theory | | 22 | | Web and wearable device. | | MVPA by wearable device:  IG: remained stable from 33.3 min/day to 33 min/day;  CG: a slight increase from 43.6 min/day to 47.4 min/day. | | Mental wellbeing (Warwick-Edinburgh Mental Wellbeing Scale), social support, pro-social behavior (Strength and Difficulties Questionnaire), and time preference for rewards: remained stable without significant changes. | | Not effective for PA promotion. | |
| Dadaczynski | | 2-arm RCT; IG: Healingo Fit, CG: no intervention control group. | | Healingo Fit is a universal prevention intervention, with the main objective to promote low levels of PA (e.g. walking activities). This was accomplished by using a tracking-based approach measuring PA with a Fitbit® pedometer (Fitbit Zip) implemented in a six-week browser-based online intervention. | | Socio-cognitive theory, theory of planned behavior, the health action process approach model | | 6 | | Web and wearable device. | | - PA-related knowledge by scale scores: IG: increased from 1.96 to 3.28, CG: remained stable; - PA-related intention by scale scores: IG: increased from 3.29 to 3.63, CG: remained stable; - PA-related self efficacy by scale scores: IG: increased from 2.65 to 3.02, CG: remained stable. | | Time spent walking per week (min/week):  IG: Increased from 401.55 to 526.48;  CG: remained stable. | | Effective for PA promotion outcomes (knowledge, intention, and self-efficacy), and walking. | |
| Direito | | 3-arm RCT; IG1: immersive app (Zombies, Run), IG2: nonimmersive app (Get Running), CG: usual behavior. | | The 2 intervention groups allowed comparison of an identical delivery approach (ie, stand-alone app), but with distinct design features: (1) use of an immersive app or (2) use of a nonimmersive app. Both apps consisted of a fully automated 8-week training program designed to improve fitness and ability to run 5 km. | | / | | 8 | | Application. | | Time to complete the 1-mile walk/run fitness test compared with CG:  IG1: decreased 28.4 seconds (*P*=.20);  IG2: decreased 24.7 sec (*P*=.32). | | No significant intervention effects were found for self-reported PA, and its perceived enjoyment, competency, autonomy, relatedness, and self-efficacy. | | Not effective for PA promotion. | |
| Edney | | 2-arm RCT; IG1: gamified version of app, IG2: nongamified version of app. | | Users in both app groups received a pedometer (Zencro, TW64S) to measure their daily step counts, and they received a weekly email with a summary of their individual step count progress. For gamified users, the email also highlighted a different app feature each week. | | Social-cognitive theory | | 14.29 | | Application. | | App attrition (30 days of nonuse):  32% of the gamified group, and 39% of the basic group. | | / | | The inclusion of gamified features  enhanced application engagement. | |
| Alexander | | 4-arm RCT; IG1: behaviorally designed gamification, IG2: loss-framed financial incentives, IG3: gamification+financial incentives, CG: attention control. | | Participants in the gamification arm were entered into a game that leverages insights from behavioral economics. In the financial incentives arm, participants were informed each week that $14 was placed in their virtual account. | | behavioral economics principles | | 48 | | Application and  wearable device. | | Step counts in post-intervention:  IG1: increased 538 steps from baseline (*P*<.01);  IG2: increased 491.8 steps from baseline (*P*<.01);  IG3: increased 868.0 steps from baseline (*P*<.01). | | Step counts in 6-month follow-up:  IG3 remained significantly greater than control group (576.2 steps, *P*<.01). | | Effective for PA promotion. | |
| Francis | | 2-arm RCT; IG: Fitbit plus MapTrek, CG: Fitbit alone. | | Players are sorted into weekly races along virtual courses and challenged to be the first to the finish line. Participants can walk whenever and wherever is convenient, steps taken are recorded by the Fitbit. All interaction with the game is done via text messages and a web-based app where they can view the course and current rankings. | | Behavior change techniques | | 24 | | Application and  wearable device. | | Daily step counts by wearable device:  IG: walked an additional 1,697 (95% CI: 996-2403) mean steps per day compared to CG. | | The number of minutes per day with at least 100 steps:  IG achieved on average 8.0 (95% CI: 4.5-11.5) additional minutes. | | Effective for PA promotion. | |
| Grade | | 2-arm crossover RCT; IG: MobileKids Monster Manor (MKMM), CG: wear the activity monitor only. | | MKMM stands out as an innovative and health-promoting mobile device-based game for youth; one where screen time is limited and physical activity is required to earn playtime and thus progress through the game. | | Self-determination theory, attribution theory | | 1 | | Application and  wearable device. | | Daily step counts by wearable device:  IG: walked an additional 2,934 mean steps per day compared to CG. | | The number of minutes per minute with at least 20 steps:  IG achieved on average 46 additional minutes compared to CG. | | Effective for PA promotion. | |
| Grade | | 2-arm RCT; IG: MobileKids Monster Manor (MKMM), CG: wear the activity monitor only. | | MKMM stands out as an innovative and health-promoting mobile device-based game for youth; one where screen time is limited and physical activity is required to earn playtime and thus progress through the game. | | Self-determination theory, attribution theory | | 2 | | Application and  wearable device. | | Daily step counts by wearable device:  IG: walked an additional 1,758 mean steps per day compared to CG. | | The number of minutes per minute with at least 20 steps:  IG achieved on average 31 additional minutes compared to CG. | | Effective for PA promotion at a short-term. | |
| Gillcrist | | 2-arm RCT; IG: game-based intervention, CG: received weekly updates. | | Participants in this study arm engaged in a game-based intervention. They were incentivized through the allocation of 70 points at the beginning of each week, which they could lose based on their performance in meeting their daily step goals. | | behavioral economics principles | | 32 | | Application and  wearable device. | | Daily step counts by wearable device:  IG: walked an additional 1,119 mean steps per day compared to CG (*P*=0.19). | | Knee Injury and Osteoarthritis Outcome Score:  IG: +0.62 compared with -0.38 in CG (*P*=0.02). | | Effective for KOA symptom improvement. | |
| Gonze | | 3-arm RCT; IG1: smartphone app, IG2: smartphone app+ tailored messages, IG3: smartphone+tailored messages+gamification, CG: usual routine. | | The intervention involves step monitoring, individual messages, and virtual challenges. For each challenge they completed, they were rewarded with a virtual badge on the app. There were rankings of the number of steps among all users, as well as running challenges. | | / | | 24 | | Application. | | Daily step counts by self-report:  IG: 3995 ± 3204 vs CG: 4250 ± 4204. | | Steps per day after rerandomization:  median -198 to +20 steps/day. | | The SMART design was feasible and changed the behavior of steps per day after rerandomization. | |
| Gremaud | | 2-arm RCT; IG: Fitbit plus MapTrek, CG: Fitbit alone. | | Players are sorted into weekly races along virtual courses and challenged to be the first to the finish line. Participants can walk whenever and wherever is convenient, steps taken are recorded by the Fitbit. All interaction with the game is done via text messages and a web-based app where they can view the course and current rankings. | | Cognitive evaluation  theory | | 10 | | Application and  wearable device. | | Daily step counts by wearable device:  IG: walked an additional 2,092 mean steps per day compared to CG. | | The number of minutes per day with at least 100 steps:  IG achieved on average 11.2 additional minutes compared to CG. | | Effective for PA promotion. | |
| Greysen | | 2-arm RCT; IG: game informed by behavioral economics, CG: feedback from wearable device. | | Intervention participants entered into a mobility game with points and levels that was run automatically, received a daily notification on their progress, and leveraged social networks. | | behavioral economics principles | | 12 | | Application and  wearable device. | | Daily step counts by wearable device:  IG did not significantly different from CG  (MD, 270 steps, *P*=.27). | | No significant difference found in functional status and urgent care utilization. | | Not effective for PA promotion. | |
| Greysen | | 2-arm RCT; IG: game informed by behavioral economics, CG: set step goals and received daily feedback. | | Intervention participants received the same feedback from the FitBit device/application and entered into an activity game with points and levels that was run automatically such that participants need only strive for step goals to play the game. | | behavioral economics principles | | 12 | | Text messages and  wearable device. | | Daily step counts:  IG was greater than CG (MD, 1699 steps/day, *P*<.01). | | Mean change in daily minutes of MVPA:  IG was greater than CG (MD, 6.6 minutes/day, *P*=.02). | | Effective for PA promotion. | |
| Haque | | 2-arm RCT; IG: mHealth app for PA motivation, CG: paper diary. | | The iGO app utilizes the 3 basic SDT psychological needs and aims to motivate users to increase their daily PA by increasing their levels of autonomy (ability to choose a daily walking task to reach a 10-minute goal after breakfast and lunch breaks), competence (feeling effective in their ongoing interactions with the social environment and to reach the daily walking goals), and relatedness (feeling connected with colleagues for the purpose of PA). | | Self-determination theory | | 4 | | Application and  wearable device. | | PA by 7-point Likert scale, mean:  IG: 6.15; CG: 4.30, *P*=.03. | | - Autonomy by 7-point Likert scale, mean: IG: 5.05; CG: 3.30, *P*<.01; - Competence by 7-point Likert scale, mean: IG: 4.86; CG: 2.00, *P*=0.01; - Relatedness by 7-point Likert scale, mean: IG: 3.15; CG: 3.43, *P*=0.54. | | Effective for PA promotion. | |
| Hochsmann | | 2-arm RCT; IG: novel PA-promoting smartphone game, CG: one-time lifestyle counselling. | | The self-developed and commercially released smartphone game “Mission: Schweinehund” was installed on the phones of participants in the intervention group. The game was designed to be self-explanatory and motivate for regular use through its game mechanics. | | Self-determination theory | | 24 | | Application. | | Intrinsic PA motivation by scale:  IG: increased 6.4±4.2, *P*<.01;  CG: decreased 1.9±16.5, *P*=.623. | | - Interest/enjoyment by scale: IG: increased 2±1.9, *P*<.01, CG: no significant changes; - Perceived competence by scale: IG: increased 2.4±2.4, *P*<.01, CG: no significant changes; - Game usage: 131.1±48.7 mins/week in-game walking, 15.3±24.6 mins/week in-game strength training.. | | Effective for PA promotion. | |
| Hochsmann | | 2-arm RCT; IG: novel PA-promoting smartphone game, CG: one-time lifestyle counselling. | | The self-developed and commercially released smartphone game “Mission: Schweinehund” was installed on the phones of participants in the intervention group. The game was designed to be self-explanatory and motivate for regular use through its game mechanics. | | Self-determination theory | | 24 | | Application. | | Daily step counts:  IG-CG: increased 3,128 steps/day, *P*<.01. | | - Aerobic capacity: Improved oxygen uptake at the first ventilatory threshold of 1.9 ml/(kg min), *P*<.001; - HbA1c: Did not change. | | Effective for PA promotion. | |
| Kurtman | | 3-arm RCT; IG1: gamification intervention, IG2: gamification intervention with PCP data sharing, CG: no intervention. | | They were entered into a game with their teammate that was designed using insights from behavioral economics to address predictable barriers to behavior change and to enhance social incentives. | | behavioral economics principles | | 36 | | Application, text messages and weight scale. | | Weight loss at 24 weeks:  IG1: -6.6 lbs, *P*<.01,  IG2: -4.8 lbs, *P*<.01,  CG: -3.9 lbs, *P*<.01. | | Weight loss at 36 weeks:  IG1: -6.3 lbs, *P*<.01,  IG2: -5.2 lbs, *P*<.01,  CG: -3.5 lbs, *P*=.01. | | Effective for weight reduction. | |
| Lee | | 2-arm RCT; IG: a gamified app, Puzzle Walk, CG: a commercial app, Google Fit. | | A gamified behavior change app, PuzzleWalk, available for both Android and Apple iOS, was developed to increase PA and reduce sedentary behavior in adults with ASD following a participatory, user-centered development process. | | Behavior change techniques | | 5 | | Application and wearable device. | | Daily step counts (mean±SE):  IG: from 5083±3092.75 to 4298.6±2699.23;  CG: from 3609.1±2849.22 to 3836.3±2486.88. | | / | | Not effective for PA promotion. | |
| Leinonen | | 2-arm RCT; IG: a gamified web-based mobile service with a wrist-worn physical activity monitor, CG: a simple activity monitor. | | The men in the intervention group had access to an automated, gamified, tailored Web-based mobile physical and social activation service during the trial. | | Transtheoretical model | | 24 | | Web and wearable device. | | Log data:  64.4% participants visited the service, 47.2% logged more than once, and 16.4% logged more than 5 times. | | Daily time spent in MVPA:  IG: increased 11.9 min, CG: decreased 9.1 min, *P*=.06. | | Not effective for PA promotion. | |
| Lewey | | 2-arm RCT; IG: gamification  intervention, CG: received daily feedback. | | Participants in the intervention arm were additionally enrolled in a team-based gamification intervention consisting of points and levels and leveraging the performance of other participants on the team. | | behavioral economics principles | | 12 | | Text messages and  wearable device. | | Daily step counts:  IG-CG: 649 steps (*P*=.009). | | Proportion of days meeting their step goal:  IG-CG: 0.11, *P*=.03. | | Effective for PA promotion. | |
| Lin | | 3-arm RCT; IG1: mobile health application-COOL Passport, IG2: Health Promotion Cloud system and game-based interactive platform with COOL Passport, CG: standard-care. | | Participants in one active intervention group were provided with COOL Passport, a mobile healthcare application. Those in the other group were provided with access to the Health Promotion Cloud system and use of game-based interactive platforms along with COOL Passport. | | Self-regulation theory | | 48 | | Application. | | Neither active group exhibited significantly greater physical activity intensity than the standard-care control group in any month during the 1 year. | | / | | Not effective for PA promotion. | |
| Maher | | 2-arm RCT; IG: online social  networking physical activity intervention, CG: wait-list control. | | Active Team is a new, free, 50-day team-based Facebook app, developed to assist adults to increase their physical activity levels. Theory of planned behavior and fun theory were selected to inform development of the content and features of Active Team. | | Theory of planned behavior, fun theory | | 7.14 | | Web and application. | | Weekly MVPA:  IG: compared with CG, increased 135 mins/week, (*P*=.03). | | Weekly walking time:  IG: compared with CG, increased 155mins/week, (*P*<.001). | | Effective for PA promotion. | |
| Mamede | | 2-arm RCT; IG: gamified digital app, CG: active control. | | Those in the intervention receiving the full version of the digital app, MoveMore, consisted of a 5-week gamification phase that included a commercially available gamified digital app incorporating several BCTs and nudges, such as social support and social comparison, followed by a physical nudges phase for the last 5 weeks. | | Behavior change techniques | | 5 | | Application and wearable device. | | Daily step counts by accelerometer:  IG: compared with CG, increased 634 (*P*=.01). | | No significant changes were observed in hours in light PA, MVPA, and sitting. | | Effective for PA promotion. | |
| Monroe | | 2-arm RCT; IG: Technology-delivered PA intervention (TECH)+gamification, CG: TECH. | | The TECH+Gamifcation group also engaged with custom-designed gamifcation elements underpinned by the SCT, self-determination theory, social network theory, and behavioral economics principles. | | Social cognitive theory, self-determination theory, social network theory, and behavioral economics principles | | 12 | | Application and wearable device. | | Daily step countsby accelerometer:  IG-CG: decreased 425 steps (*P*=.19). | | MVPA by accelerometer:  IG-CG: decreased 3.36 min/day (*P*=.21). | | Not effective for PA promotion. | |
| Nishiwaki | | 2-arm crossover RCT; IG: game intervention, CG: normal activity monitor. | | The participants wore both a normal activity monitor and an activity monitor with computerized game functions during the game intervention period. | | / | | 12 | | Activity monitor with game function. | | Daily step counts by activity monitor:  IG vs CG: 10,520 ± 562 vs 8,711±523 (*P*<.01). | | Amount of PA (METs h/day) by activity monitor:  IG vs CG: 3.1±0.2 vs 2.4±0.2 (*P*<.01). | | Effective for PA promotion. | |
| Nuijten | | 2-arm RCT; IG: personalized complexity parameters, CG: general complexity parameters. | | GameBus was especially designed for health promotion and provides a highly configurable gamification engine that is used for sustaining participant engagement. | | / | | 8 | | Application. | | Personalization was effective for promoting physical activity frequency. | | / | | Personalization seems particularly promising for promoting the frequency of physical activity. | |
| Patel | | 2-arm RCT; IG: gamification intervention plus daily feedback, CG: daily feedback control. | | Participants in the gamification arm were entered into a game with their family for 12 weeks that was designed using insights from behavioral economics to address predictable barriers to behavior change and to enhance social incentives. | | behavioral economics principles | | 12 | | Application, text messages and wearable device. | | Daily step counts:  IG-CG: MD 953 steps/day, (*P*<.001). | | Proportion of days meeting their step goal:  IG-CG: Increased 0.27 participant-day (*P*<.001). | | Effective for PA promotion. | |
| Patel | | 4-arm RCT; IG1: support gamification, IG2: collaboration gamification, IG3: competition gamification, CG: regular feedback control. | | Participants in the intervention arms were entered into a game with points and levels that was run automatically and provided a daily notification of their progress. | | behavioral economics principles | | 24 | | Application, text messages and wearable device. | | Daily step counts:  IG1-CG: MD 689 steps/day (*P*<.001);  IG2-CG: MD 637 steps/day (*P*=.001);  IG3-CG: MD 920 steps/day (*P*<.001). | | Proportion of days meeting their step goal:  IG1-CG: Increased 0.11 participant-day (*P*<.001);  IG2-CG: Increased 0.11 participant-day (*P*<.001);  IG3-CG: Increased 0.16 participant-day (*P*<.001). | | Effective for PA promotion. | |
| Patel | | 5-arm RCT; IG1: gamification with assigned and immediate goals, IG2: gamification with assigned and gradual goals, IG3: gamification with choice and immediate goals, IG4: gamification with choice and gradual goals, CG: daily feedback control. | | Participants in the gamification arms were randomly assigned to have step goals either assigned or self-chosen. Participants in the self-chosen arms could change their goal at any time during the study as long as it was within the provided range. When the study began, goals were implemented either immediately or gradually over the 8-week introductory period. | | behavioral economics principles | | 16 | | Application, text messages and wearable device. | | Daily step counts at week 9 to 16:  IG1-CG: MD 523 steps/day (*P*=.08);  IG2-CG: MD 616 steps/day (*P*=.04);  IG3-CG: MD 1384 steps/day (*P*<.001);  IG4-CG: MD 569 steps/day (*P*=.05). | | Daily step counts at week 17 to 24:  IG1-CG: MD 473 steps/day (*P*=.12);  IG2-CG: MD 776 steps/day (*P*=.01);  IG3-CG: MD 1391 steps/day (*P*<.001);  IG4-CG: MD 578 steps/day (*P*=.06). | | Effective for PA promotion. | |
| Patel | | 4-arm RCT; IG1: support gamification, IG2: collaboration gamification, IG3: competition gamification, CG: goal setting and regular feedback control. | | Participants in the intervention arms were entered into a game with points and levels that ran automatically and provided a daily notification on their progress. | | behavioral economics principles | | 48 | | Application, text messages and wearable device. | | Daily step counts:  IG1-CG: MD 503 steps/day (*P*=.01);  IG2-CG: MD 280 steps/day (*P*=.16);  IG3-CG: MD 606 steps/day (*P*=.003). | | / | | Effective for PA promotion for support and competition group. | |
| Pyky | | 2-arm RCT; IG: MOPOrtal service, CG: received wearable device. | | The intervention group was given access to a novel mobile service (MOPOrtal). The aim of the MOPOrtal was to motivate participants physically, mentally and socially. In addition, the participants in the intervention group received tailored feedback. | | Transtheoretical model | | 24 | | Web and  wearable device. | | Life satisfaction:  IG: improved (*P*<.001), CG: improved (*P*=.01). | | No significant changes were observed in self-rated health. | | Effective for life satisfaction improvement. | |
| Tu | | 2-arm RCT; IG1: Walkup app, IG2: WeChat Sports app. | | Walkup and WeChat Sports were selected because these two fitness apps are distinct enough to represent different gamification orientations: Walkup includes five commonly used game elements that aim at enhancing enjoyment, and has few social interaction elements; in contrast, WeChat Sport includes three game elements that are commonly applied to enhance social interaction, but has none of the enjoyment-enhancing elements that Walkup has. | | / | | 7 | | Application. | | Daily step counts on the last day (mean±SD):  IG1: Walkup 4737.32±5446.46;  IG2: WeChat Sports 8775.80±5421.74. | | Intentions to continue exercising (mean±SD):  IG1: 2.97±1.11;  IG2: 3.76±0.81. | | Compared to make it fun, making it more social can be more effective in helping  them sustain a walking routine in their daily living. | |
| Robertson | | 2-arm RCT; IG: FitQuest app, CG: standard PE lesson. | | The most recent version of the system runs on the Android operating system, utilising GPS technology and Google MapsTM in order to provide a series of 8 exercise based mini-games. | | / | | 5 | | Application. | | Self-efficacy by scale:  IG: from 24.7±4.1 to 25.8 ±4.1;  CG: from 24.9±4.4 to 24.8±4.1. | | Step count:  IG: from 6033±1809 to 5213±1692;  CG: from 6287±1414 to 6081±1945. | | Not effective for PA promotion. | |
| Robertson | | 2-arm RCT; IG: multimedia messaging service messages plus activity tracker, CG: received physical activity tracker. | | The participants in the experimental group received Steps2Health multimedia messaging service (MMS) messages. These messages presented information about a virtual journey through Japan’s Inland Sea region and were triggered by step counts in real time. | | Self-determination theory | | 4 | | Web, application and wearable device. | | Motivation for physical activity:  Both groups improved (*P*=.04). | | - Self-reported leisure activity score: IG improved (*P*=.005); - Daily step counts: IG improved (*P*=.02). | | Effective for PA promotion. | |
| Santos | | 2-arm RCT; IG: social pervasive game, CG: pervasive game without social interaction. | | Participants played a pervasive location-based mobile game called Shinpo, which, in free translation, means “steps of the gods” in Japanese. | | / | | 4 | | Application. | | Weekly step counts changes to baseline values (mean±SE):  IG: 7648±3900.9;  CG: -1041.8±1992.7. | | / | | Effective for PA promotion. | |
| Sanudo | | 2-arm RCT; IG: gamified mHealth intervention, CG: control condition without gamification. | | Study was designed with a specific aim to examine the effects of gamification by modifying social comparison. Additionally, it is essential to recognize that the intervention also inherently incorporated other behavior change techniques, grounded in established theories. | | Behavior change techniques | | 8 | | Application and wearable device. | | - Step counts by accelerometer: IG: from 8278.8±4073.8 to 11895.7±4976.0, *P*<0.001; CG: from 7580.0±3937.6 to 12203±4711.6, *P*<0.001. - MVPA (min/weeks) by accelerometer: IG: from 119.6±56 to 164.4±79.3, *P*=0.005; CG: from 98.7±50.4 to 163.3±61.5, *P*<0.001. | | Sleep hours (h/day) by accelerometer:  IG: from 7.6±1.0 to 7.8±1.0, *P*=0.56;  CG: from 7.7±1.6 to 7.5±1.9, *P*=0.54. | | Not effective for PA promotion regarding the addition of gamified social comparison. | |
| Schwarz | | 2-arm RCT; IG: dynamically tailored exergame, CG: non-tailored exergame. | | The study is part of the European SmartLife project which aims to increase physical activity among adolescents using a mobile exergame. The game developed for this project consisted of a narrative-driven, single player, audio-supported mobile exergame. | | / | | 4 | | Application and  accelerometer T-shirt. | | Overall kids game experience questionnaire:  IG: 2.03; CG: 1.97, *P*=0.62. | | / | | Not effective for game engagement improvement. | |
| Simmering | | 2-arm RCT; IG: VA MapTrek, CG: received a Fitbit activity tracker. | | Half of the veterans were assigned to participate in VA MapTrek, a text-message-based race game designed to increase step counts. | | / | | 8 | | Application and wearable device. | | Step counts by fitbit:  IG have taken an extra 96,627 steps (equivalent to 48.3 additional miles) than CG. | | / | | Effective for PA promotion. | |
| Thorsteinsen | | 2-arm RCT; IG: Lifestyle intervention, CG: no intervention. | | The intervention Lifestyle Tool consisted of a rule-based website designed to help people plan and monitor their physical activity in order to become more physically active. | | / | | 12 | | Web. | | - Minutes exercising at week 5: IG: 576.67±235.29, CG: 413.13±224.42; - Minutes exercising at week 9: IG: 660.25±359.60, CG: 377.25±167.39; - Minutes exercising at week 12: IG: 574.42±434.74, CG: 501.88±232.90. | | / | | Effective for PA promotion at a short-term. | |
| Waddell | | 2-arm RCT; IG: gamification with social incentives, CG: received wearable device feedback. | | The gamification arm engaged in an 8-week game with loss-framed points and levels to help achieve their step goal and received daily (text message) and weekly (email) feedback describing their progress. | | behavioral economics principles | | 8 | | Text messages and  wearable device. | | Daily step counts:  IG-CG: MD 981 steps/day, *P*=.01. | | Proportion of days meeting their step goal:  IG-CG: Increased 0.41 participant-day (*P*<.001). | | Effective for PA promotion. | |
| Xu | | 3-arm RCT; IG1: individual gamified intervention, IG2: team gamified intervention, CG: received daily goal setting. | | Patients in the individual and team groups received the  gamification intervention based on behavioral economics  principles via the WeChat applet. | | behavioral economics principles | | 12 | | Application. | | Daily step counts:  IG1-CG: MD 988 steps/day, *P*<.01;  IG2-CG: MD 472 steps/day, *P*=.12. | | Proportion of days meeting their step goal:  IG1-CG: Increased 0.08 participant-day (*P*<.001);  IG2-CG: Increased 0.04 participant-day (*P*=.074). | | Effective for PA promotion in IG1. | |
| Zuckerman | | 3-arm RCT; IG1: points version of StepByStep, IG2: leaderboard version, CG: quantified version. | | The study evaluated the effectiveness of two game elements: virtual rewards and social comparison. | | / | | 2 | | Application. | | Daily walking time:  IG1: 49.94±24.78, IG2: 40.85±18.40, CG: 43.63±20.36. | | / | | Effective for PA promotion in points version. | |
| Maria do | | 3-arm RCT; IG1: smartphone app, IG2: smartphone app+ tailored messages, IG3: smartphone+tailored messages+gamification, CG: usual routine. | | The intervention involves step monitoring, individual messages, and virtual challenges. For each challenge they completed, they were rewarded with a virtual badge on the app. There were rankings of the number of steps among all users, as well as running challenges. | | / | | 24 | | Application. | | Daily step counts by self-report: IG: 3995 ± 3204 vs CG: 4250 ± 4204. | | Steps per day after rerandomization: median -198 to +20 steps/day. | | The SMART design was feasible and changed the behavior of steps per day after rerandomization. | |
| Patel | | 2-arm RCT; IG: gamification+automated coaching, CG: attention control. | | All participants received a Fitbit, selected a step-count goal, and were advised to undertake home-based walking. IG participants entered a 16-week automated loss-framed game with weekly points, 5 levels, fresh starts, and a support partner; they also received biweekly automated coaching text messages. | | Behavioral economics principles | | 16 | | Text messages and wearable device. | | Daily step counts during weeks 5-16: IG-CG: +920 steps/day (95% CI -21 to 1861; P=.06). | | Daily step counts during weeks 17-24: IG-CG: +1074 steps/day (95% CI 133 to 2015; *P*=.03). | | Gamification plus automated coaching increased PA compared with attention control over the 24-week study, with a significant effect during follow-up. | |
| Patel | | 2-arm RCT; IG: behaviorally designed gamification, CG: attention control. | | All participants received a Fitbit, established a baseline step count, and selected a step goal increase. IG participants entered a 24-week automated game using a precommitment pledge, weekly loss-framed points, levels, fresh starts, a support partner, and a nominal trophy. | | Behavioral economics principles | | 24 | | Text messages and wearable device. | | Daily step counts during the 24-week intervention: IG-CG: +759 steps/day (95% CI 209 to 1309; *P*=.007). | | Weekly MVPA during intervention: IG-CG +16 min/week (95% CI 4 to 29; *P*=.010); during 12-week follow-up: +11 min/week (95% CI 0 to 22; *P*=.048). Daily steps during follow-up: +581 steps/day (*P*=.070). | | Behaviorally designed gamification increased PA among Black and Hispanic breast and prostate cancer survivors. | |
| Janis | | 2-arm cluster RCT; IG: SMARTFAMILY2.0 app, CG: waiting-list control. | | Families received study smartphones with the SMARTFAMILY2.0 app and Bluetooth-linked accelerometers. They set collective weekly goals for steps, MVPA, joint family PA, fruit and vegetable intake, and shared meals. The app provided an animated tailored coach, points/stars and badges, personalized progress feedback, leaderboards and peer support, weekly goal prompts, and just-in-time prompts after prolonged inactivity. | | Self-determination theory and behavior change techniques; just-in-time adaptive intervention. | | 3 | | Application and wearable device. | | Primary outcomes: Device-measured MVPA favored the CG at T1 (β = −.50, *P*=.02); no significant effects were found for step count or self-reported MVPA. | | Secondary outcomes: Joint PA favored the IG at T1 and T2. | | Not effective for increasing overall PA in an already active sample. | |
| Nimet | | 2-arm single-blind cluster RCT; IG: gamified T2M Android mobile application; CG: nongamified T2M Android mobile application. | | Students used the T2M Android app to pursue a daily goal of 10,000 steps and complete exercise goals. Daily step counts were measured with a pedometer and uploaded to the app, while exercise videos were viewed and participants recorded their exercise performance. The IG additionally received points, digital step and exercise badges, a leaderboard, and achievement certificates and medals. | | Self-determination theory. | | 4 | | Application and pedometer. | | Daily goal completion favored the IG: step-goal completion was 65% versus 40% in the CG (*P*=.043, d=.40), and exercise-goal completion was 68% versus 15% (*P*=.0001, d=.77). | | PAQ-C scores improved more in the IG than in the CG (*P*=.0114, d=.51). At 6 months, 70% of the IG versus 27% of the CG reported continuing to exercise. | | The gamified T2M app increased completion of daily step and exercise goals and improved self-reported physical activity among sedentary secondary school students, with some maintenance of exercise behavior at 6 months. | |
| Gaizka | | 2-arm single-center pragmatic RCT; IG: gamified family-based online exercise platform; CG: routine care. | | Children completed a 12-week family-based exercise program through an online gamified platform at home or outdoors, with parental supervision. Two sessions were available each week and included warm-up, bodyweight strength, cardiorespiratory, color-coded, and cooldown exercises. Exercises increased progressively in length and intensity. The platform included points, a calendar/session guide, customizable avatars, 14 unlockable worlds, novice-to-expert levels, virtual accessories and other milestone-contingent rewards. Platform use and session completion were monitored weekly, and nonadherent families received telephone encouragement and support. | | / | | 12 | | Web-based online gamified exercise platform. | | No significant between-group effect was found for accelerometer-measured MVPA after 12 weeks. | | The IG showed greater improvements in light PA (+26.44 min/day, *P*=.01), total PA (+30.88 min/day, *P*=.02), standing long jump (+12.26 cm, *P*<.001), and overall physical fitness z-score (+0.15, *P*=.04). Other movement, fitness, motor competence, and executive-function outcomes did not differ significantly. | | The gamified family-based intervention improved light and total physical activity and selected physical fitness outcomes, but did not significantly improve the primary MVPA outcome. | |
| Jeffrey Z | | 4-arm parallel RCT; SG: gamification elements introduced one at a time; CG: the same elements introduced sequentially and retained cumulatively; EG: commercial mobile walking game; control: tracking only. | | All intervention groups selected an individualized daily step goal. SG received, in sequence, points with weekly progress feedback, daily leaderboards with recognition of the top 3 participants, team-based social interaction and step-count competition through LINE OpenChat, and Pikmin Bloom. CG received the same elements, but each remained active after introduction. EG played Pikmin Bloom throughout the intervention; the game linked real-world steps to points and levels and included avatars, virtual character collection, location-based exploration, social collaboration, and community challenges. | | / | | 7 | | Applications. | | Daily step count by smartphone app-based self-report: all gamification arms increased steps versus control during intervention; cumulative delivery produced the largest effect (+2,326 steps/day, *P*<.001). At follow-up, effects remained significant for cumulative delivery (+1,284/day, *P*=.005) and explicit gamification (+1,027/day, *P*=.025), but not sequential delivery. | | No consistent biometric effects were found; resting heart rate improved transiently in SG and systolic blood pressure improved transiently in CG, but these effects were not maintained at follow-up. | | Cumulative delivery of gamification elements was the most effective strategy for increasing daily steps. Explicit gamification showed a slower but more durable pattern, whereas the effect of sequential delivery was not sustained after intervention withdrawal. | |
| Alexandre | | 2-arm parallel RCT; IG: Kiplin digital gamified adapted physical activity program; CG: usual-care face-to-face supervised adapted physical activity program. | | The 12-week Kiplin intervention comprised 2 initial face-to-face sessions, 20 live group telecoaching sessions, 3 collective mobile-app games, an in-app chat, and real-time activity monitoring linked to a Garmin wearable. Daily steps were converted into points and used to advance through game narratives: collectively reaching destinations on a world map, completing challenges to unlock clues and solve missions, and progressing through levels in a forest-rescue board game. | | Social identity approach and self-determination theory. | | 12 | | Application and wearable device. | | Wearable-measured daily steps: compared with usual care, the Kiplin group showed a greater increase from baseline during the 3-month intervention (+1085 steps/day; 95% CI 493-1676; *P*<.001) and the 6-month postintervention follow-up (+1775 steps/day; 95% CI 906-2643; *P*<.001). | | MVPA improved in favor of the Kiplin group (group×time β=5.69, 95% CI 0.61-10.77; *P*=.03). No significant group×time effects were found for the other clinical outcomes, and cost-utility did not differ significantly between programs. | | The digital gamified Kiplin program increased and sustained daily physical activity compared with usual care, although responses varied substantially between individuals and no clear clinical or economic superiority was demonstrated. | |
| B. Sañudo | | 2-arm multicentre pilot RCT; IG: IDHEApp gamified mHealth intervention plus Fitbit Charge 6; CG: usual routine with Fitbit Charge 6 used for measurement only. | | The 8-week IDHEApp intervention used Fitbit Charge 6 data and delivered daily personalised challenges targeting physical activity, sedentary behavior, and nutrition. Participants earned points, received digital badges for behavioral milestones, viewed visual progress indicators, received notifications and feedback, and could access overall and behavior-specific leaderboards. | | / | | 8 | | Application and wearable device. | | Daily steps: the intervention group had a significantly greater post-intervention step count than the control group after baseline adjustment (adjusted mean difference 2326.72 steps/day, 95% CI 763.97-3461.23; *P*=.001; Cohen d=0.97). | | No significant between-group effects were found for light, moderate, or vigorous physical activity, sitting time, or standing time. | | The pilot RCT suggests that an accessible gamified mHealth intervention is feasible and may increase daily ambulatory activity among young people with intellectual disabilities, although evidence for other activity intensities and sedentary behavior remained exploratory. | |
| Ming | | 2-arm parallel-group RCT; IG: gamified team-based mHealth intervention; CG: active technology-based PA monitoring. | | Both groups used the Shouti Fitness app and a fitness watch, with daily targets of ≥30 min MVPA or ≥180 MET-min and weekly targets of ≥150 min MVPA or ≥900 MET-min. The IG was additionally assigned to 4 teams and received rank-based points, top-performer bonus points, real-time MVPA/MET-min progress bars, midweek champion updates, weekly team and individual leaderboards, cumulative volunteer hours, honorary certificates, academic-credit coordination, and a 400-Chinese-yuan material reward for top-10% performers. | | Self-determination theory and theory of planned behavior. | | 8 | | Application and wearable device. | | Across 8 weeks, IG versus CG: daily steps 10,356±1,245 versus 8,242±1,087 (Δ=2,114; d=1.81); daily MVPA 71±15 versus 43±12 min (Δ=28; d=2.06); weekly MET-min 1,650±310 versus 1,340±285 (Δ=310; d=1.04); goal completion 88.9%±8.5 versus 77.5%±10.2 and data synchronization 89.1%±7.7 versus 75.5%±11.3 (all *P*<.001). | | Significant effects favored the IG for skeletal muscle (+0.54 kg), body fat (−0.46 percentage points), basal metabolic rate (+7.8 kcal/day), 800/1000-m run time (−6.5 s), vital capacity (+190 mL), sit-and-reach (+1.8 cm), female sit-ups (+4.5), cognitive flexibility (−29 ms), and depressive symptoms (−2.0 points). | | Adding team-based gamification and incentives to an otherwise identical wearable-linked mHealth PA platform substantially improved PA, adherence, and selected physical, cognitive, and mental health outcomes among college students; no serious adverse events occurred. | |
| Table S2. Intervention components, theoretical foundations, delivery systems, intervention duration (time frame), and effects on primary and secondary outcomes for the 53 included randomized controlled trials.  Abbreviation: PA, physical activity; RCT, randomized controlled trials; IG, intervention group; CG, control group; MVPA, moderate to vigorous physical activity; SDT, self-determination theory; PCP, primary care physician; ASD, autism spectrum disorder; SE, standard error; COOL, care & organize our lifestyle; BCT, behavior change technique; MD, mean difference. | | | | | | | | | | | | | | | | |  |
| Author | Study arms | | Intervention contents | | Theoretical basis | | Duration (weeks) | | Type of systems | | Primary outcome | | Secondary outcome | | Conclusion | |  |
| Agarwal | 3-arm RCT; IG1: gamification with social support, IG2: gamification with social support and loss-framed financial incentives, CG: feedback only. | | All participants received a wearable device to track step counts and selected a step goal. Participants in the 2 gamification groups were entered into a 12-week game. | | behavioral economics principles | | 12 | | Application and wearable device. | | Mean daily steps by wearable device:  IG1-CG: Increased 433 steps (*P* = .81);  IG2-CG: Increased 1224 steps (*P* = .005). | | Proportion of days meeting their step goal:  IG1-CG: Increased 0.21 participant-day (*P* < .001);  IG2-CG: Increased 0.34 participant-day (*P* < .001). | | Effective for promoting physical activity in IG2 but not in IG1. | |  |
| Corepal [26] | 2-arm RCT; IG: The StepSmart Challenge, CG: no intervention control group. | | The StepSmart Challenge used gamification strategies, combined with the core tenets of self-determination theory, to encourage and support physical activity behavior change by moving participants along the motivation continuum from more external forms of motivation towards intrinsic motivation. | | Self-determination theory | | 22 | | Web and wearable device. | | MVPA by wearable device:  IG: remained stable from 33.3 min/day to 33 min/day;  CG: a slight increase from 43.6 min/day to 47.4 min/day. | | Mental wellbeing (Warwick-Edinburgh Mental Wellbeing Scale), social support, pro-social behavior (Strength and Difficulties Questionnaire), and time preference for rewards: remained stable without significant changes. | | Not effective for PA promotion. | |  |
| Dadaczynski [27] | 2-arm RCT; IG: Healingo Fit, CG: no intervention control group. | | Healingo Fit is a universal prevention intervention, with the main objective to promote low levels of PA (e.g. walking activities). This was accomplished by using a tracking-based approach measuring PA with a Fitbit® pedometer (Fitbit Zip) implemented in a six-week browser-based online intervention. | | Socio-cognitive theory, theory of planned behavior, the health action process approach model | | 6 | | Web and wearable device. | | - PA-related knowledge by scale scores: IG: increased from 1.96 to 3.28, CG: remained stable; - PA-related intention by scale scores: IG: increased from 3.29 to 3.63, CG: remained stable; - PA-related self efficacy by scale scores: IG: increased from 2.65 to 3.02, CG: remained stable. | | Time spent walking per week (min/week):  IG: Increased from 401.55 to 526.48;  CG: remained stable. | | Effective for PA promotion outcomes (knowledge, intention, and self-efficacy), and walking. | |  |
| Direito [28] | 3-arm RCT; IG1: immersive app (Zombies, Run), IG2: nonimmersive app (Get Running), CG: usual behavior. | | The 2 intervention groups allowed comparison of an identical delivery approach (ie, stand-alone app), but with distinct design features: (1) use of an immersive app or (2) use of a nonimmersive app. Both apps consisted of a fully automated 8-week training program designed to improve fitness and ability to run 5 km. | | / | | 8 | | Application. | | Time to complete the 1-mile walk/run fitness test compared with CG:  IG1: decreased 28.4 seconds (*P*=.20);  IG2: decreased 24.7 sec (*P*=.32). | | No significant intervention effects were found for self-reported PA, and its perceived enjoyment, competency, autonomy, relatedness, and self-efficacy. | | Not effective for PA promotion. | |  |
| Edney [29] | 2-arm RCT; IG1: gamified version of app, IG2: nongamified version of app. | | Users in both app groups received a pedometer (Zencro, TW64S) to measure their daily step counts, and they received a weekly email with a summary of their individual step count progress. For gamified users, the email also highlighted a different app feature each week. | | Social-cognitive theory | | 14.29 | | Application. | | App attrition (30 days of nonuse):  32% of the gamified group, and 39% of the basic group. | | / | | The inclusion of gamified features  enhanced application engagement. | |  |
| Alexander [30] | 4-arm RCT; IG1: behaviorally designed gamification, IG2: loss-framed financial incentives, IG3: gamification+financial incentives, CG: attention control. | | Participants in the gamification arm were entered into a game that leverages insights from behavioral economics. In the financial incentives arm, participants were informed each week that $14 was placed in their virtual account. | | behavioral economics principles | | 48 | | Application and  wearable device. | | Step counts in post-intervention:  IG1: increased 538 steps from baseline (*P*<.01);  IG2: increased 491.8 steps from baseline (*P*<.01);  IG3: increased 868.0 steps from baseline (*P*<.01). | | Step counts in 6-month follow-up:  IG3 remained significantly greater than control group (576.2 steps, *P*<.01). | | Effective for PA promotion. | |  |
| Francis [31] | 2-arm RCT; IG: Fitbit plus MapTrek, CG: Fitbit alone. | | Players are sorted into weekly races along virtual courses and challenged to be the first to the finish line. Participants can walk whenever and wherever is convenient, steps taken are recorded by the Fitbit. All interaction with the game is done via text messages and a web-based app where they can view the course and current rankings. | | Behavior change techniques | | 24 | | Application and  wearable device. | | Daily step counts by wearable device:  IG: walked an additional 1,697 (95% CI: 996-2403) mean steps per day compared to CG. | | The number of minutes per day with at least 100 steps:  IG achieved on average 8.0 (95% CI: 4.5-11.5) additional minutes. | | Effective for PA promotion. | |  |
| Garde [33] | 2-arm crossover RCT; IG: MobileKids Monster Manor (MKMM), CG: wear the activity monitor only. | | MKMM stands out as an innovative and health-promoting mobile device-based game for youth; one where screen time is limited and physical activity is required to earn playtime and thus progress through the game. | | Self-determination theory, attribution theory | | 1 | | Application and  wearable device. | | Daily step counts by wearable device:  IG: walked an additional 2,934 mean steps per day compared to CG. | | The number of minutes per minute with at least 20 steps:  IG achieved on average 46 additional minutes compared to CG. | | Effective for PA promotion. | |  |
| Garde [32] | 2-arm RCT; IG: MobileKids Monster Manor (MKMM), CG: wear the activity monitor only. | | MKMM stands out as an innovative and health-promoting mobile device-based game for youth; one where screen time is limited and physical activity is required to earn playtime and thus progress through the game. | | Self-determination theory, attribution theory | | 2 | | Application and  wearable device. | | Daily step counts by wearable device:  IG: walked an additional 1,758 mean steps per day compared to CG. | | The number of minutes per minute with at least 20 steps:  IG achieved on average 31 additional minutes compared to CG. | | Effective for PA promotion at a short-term. | |  |
| Gillcrist [34] | 2-arm RCT; IG: game-based intervention, CG: received weekly updates. | | Participants in this study arm engaged in a game-based intervention. They were incentivized through the allocation of 70 points at the beginning of each week, which they could lose based on their performance in meeting their daily step goals. | | behavioral economics principles | | 32 | | Application and  wearable device. | | Daily step counts by wearable device:  IG: walked an additional 1,119 mean steps per day compared to CG (*P*=0.19). | | Knee Injury and Osteoarthritis Outcome Score:  IG: +0.62 compared with -0.38 in CG (*P*=0.02). | | Effective for KOA symptom improvement. | |  |
| Gonze [35] | 3-arm RCT; IG1: smartphone app, IG2: smartphone app+ tailored messages, IG3: smartphone+tailored messages+gamification, CG: usual routine. | | The intervention involves step monitoring, individual messages, and virtual challenges. For each challenge they completed, they were rewarded with a virtual badge on the app. There were rankings of the number of steps among all users, as well as running challenges. | | / | | 24 | | Application. | | Daily step counts by self-report:  IG: 3995 ± 3204 vs CG: 4250 ± 4204. | | Steps per day after rerandomization:  median -198 to +20 steps/day. | | The SMART design was feasible and changed the behavior of steps per day after rerandomization. | |  |
| Gremaud [36] | 2-arm RCT; IG: Fitbit plus MapTrek, CG: Fitbit alone. | | Players are sorted into weekly races along virtual courses and challenged to be the first to the finish line. Participants can walk whenever and wherever is convenient, steps taken are recorded by the Fitbit. All interaction with the game is done via text messages and a web-based app where they can view the course and current rankings. | | Cognitive evaluation  theory | | 10 | | Application and  wearable device. | | Daily step counts by wearable device:  IG: walked an additional 2,092 mean steps per day compared to CG. | | The number of minutes per day with at least 100 steps:  IG achieved on average 11.2 additional minutes compared to CG. | | Effective for PA promotion. | |  |
| Greysen [37] | 2-arm RCT; IG: game informed by behavioral economics, CG: feedback from wearable device. | | Intervention participants entered into a mobility game with points and levels that was run automatically, received a daily notification on their progress, and leveraged social networks. | | behavioral economics principles | | 12 | | Application and  wearable device. | | Daily step counts by wearable device:  IG did not significantly different from CG  (MD, 270 steps, *P*=.27). | | No significant difference found in functional status and urgent care utilization. | | Not effective for PA promotion. | |  |
| Greysen [38] | 2-arm RCT; IG: game informed by behavioral economics, CG: set step goals and received daily feedback. | | Intervention participants received the same feedback from the FitBit device/application and entered into an activity game with points and levels that was run automatically such that participants need only strive for step goals to play the game. | | behavioral economics principles | | 12 | | Text messages and  wearable device. | | Daily step counts:  IG was greater than CG (MD, 1699 steps/day, *P*<.01). | | Mean change in daily minutes of MVPA:  IG was greater than CG (MD, 6.6 minutes/day, *P*=.02). | | Effective for PA promotion. | |  |
| Haque [39] | 2-arm RCT; IG: mHealth app for PA motivation, CG: paper diary. | | The iGO app utilizes the 3 basic SDT psychological needs and aims to motivate users to increase their daily PA by increasing their levels of autonomy (ability to choose a daily walking task to reach a 10-minute goal after breakfast and lunch breaks), competence (feeling effective in their ongoing interactions with the social environment and to reach the daily walking goals), and relatedness (feeling connected with colleagues for the purpose of PA). | | Self-determination theory | | 4 | | Application and  wearable device. | | PA by 7-point Likert scale, mean:  IG: 6.15; CG: 4.30, *P*=.03. | | - Autonomy by 7-point Likert scale, mean: IG: 5.05; CG: 3.30, *P*<.01; - Competence by 7-point Likert scale, mean: IG: 4.86; CG: 2.00, *P*=0.01; - Relatedness by 7-point Likert scale, mean: IG: 3.15; CG: 3.43, *P*=0.54. | | Effective for PA promotion. | |  |
| Hochsmann [40] | 2-arm RCT; IG: novel PA-promoting smartphone game, CG: one-time lifestyle counselling. | | The self-developed and commercially released smartphone game “Mission: Schweinehund” was installed on the phones of participants in the intervention group. The game was designed to be self-explanatory and motivate for regular use through its game mechanics. | | Self-determination theory | | 24 | | Application. | | Intrinsic PA motivation by scale:  IG: increased 6.4±4.2, *P*<.01;  CG: decreased 1.9±16.5, *P*=.623. | | - Interest/enjoyment by scale: IG: increased 2±1.9, *P*<.01, CG: no significant changes; - Perceived competence by scale: IG: increased 2.4±2.4, *P*<.01, CG: no significant changes; - Game usage: 131.1±48.7 mins/week in-game walking, 15.3±24.6 mins/week in-game strength training.. | | Effective for PA promotion. | |  |
| Hochsmann [41] | 2-arm RCT; IG: novel PA-promoting smartphone game, CG: one-time lifestyle counselling. | | The self-developed and commercially released smartphone game “Mission: Schweinehund” was installed on the phones of participants in the intervention group. The game was designed to be self-explanatory and motivate for regular use through its game mechanics. | | Self-determination theory | | 24 | | Application. | | Daily step counts:  IG-CG: increased 3,128 steps/day, *P*<.01. | | - Aerobic capacity: Improved oxygen uptake at the first ventilatory threshold of 1.9 ml/(kg min), *P*<.001; - HbA1c: Did not change. | | Effective for PA promotion. | |  |
| Kurtman [42] | 3-arm RCT; IG1: gamification intervention, IG2: gamification intervention with PCP data sharing, CG: no intervention. | | They were entered into a game with their teammate that was designed using insights from behavioral economics to address predictable barriers to behavior change and to enhance social incentives. | | behavioral economics principles | | 36 | | Application, text messages and weight scale. | | Weight loss at 24 weeks:  IG1: -6.6 lbs, *P*<.01,  IG2: -4.8 lbs, *P*<.01,  CG: -3.9 lbs, *P*<.01. | | Weight loss at 36 weeks:  IG1: -6.3 lbs, *P*<.01,  IG2: -5.2 lbs, *P*<.01,  CG: -3.5 lbs, *P*=.01. | | Effective for weight reduction. | |  |
| Lee [43] | 2-arm RCT; IG: a gamified app, Puzzle Walk, CG: a commercial app, Google Fit. | | A gamified behavior change app, PuzzleWalk, available for both Android and Apple iOS, was developed to increase PA and reduce sedentary behavior in adults with ASD following a participatory, user-centered development process. | | Behavior change techniques | | 5 | | Application and wearable device. | | Daily step counts (mean±SE):  IG: from 5083±3092.75 to 4298.6±2699.23;  CG: from 3609.1±2849.22 to 3836.3±2486.88. | | / | | Not effective for PA promotion. | |  |
| Leinonen [44] | 2-arm RCT; IG: a gamified web-based mobile service with a wrist-worn physical activity monitor, CG: a simple activity monitor. | | The men in the intervention group had access to an automated, gamified, tailored Web-based mobile physical and social activation service during the trial. | | Transtheoretical model | | 24 | | Web and wearable device. | | Log data:  64.4% participants visited the service, 47.2% logged more than once, and 16.4% logged more than 5 times. | | Daily time spent in MVPA:  IG: increased 11.9 min, CG: decreased 9.1 min, *P*=.06. | | Not effective for PA promotion. | |  |
| Lewey [45] | 2-arm RCT; IG: gamification  intervention, CG: received daily feedback. | | Participants in the intervention arm were additionally enrolled in a team-based gamification intervention consisting of points and levels and leveraging the performance of other participants on the team. | | behavioral economics principles | | 12 | | Text messages and  wearable device. | | Daily step counts:  IG-CG: 649 steps (*P*=.009). | | Proportion of days meeting their step goal:  IG-CG: 0.11, *P*=.03. | | Effective for PA promotion. | |  |
| Lin [46] | 3-arm RCT; IG1: mobile health application-COOL Passport, IG2: Health Promotion Cloud system and game-based interactive platform with COOL Passport, CG: standard-care. | | Participants in one active intervention group were provided with COOL Passport, a mobile healthcare application. Those in the other group were provided with access to the Health Promotion Cloud system and use of game-based interactive platforms along with COOL Passport. | | Self-regulation theory | | 48 | | Application. | | Neither active group exhibited significantly greater physical activity intensity than the standard-care control group in any month during the 1 year. | | / | | Not effective for PA promotion. | |  |
| Maher [47] | 2-arm RCT; IG: online social  networking physical activity intervention, CG: wait-list control. | | Active Team is a new, free, 50-day team-based Facebook app, developed to assist adults to increase their physical activity levels. Theory of planned behavior and fun theory were selected to inform development of the content and features of Active Team. | | Theory of planned behavior, fun theory | | 7.14 | | Web and application. | | Weekly MVPA:  IG: compared with CG, increased 135 mins/week, (*P*=.03). | | Weekly walking time:  IG: compared with CG, increased 155mins/week, (*P*<.001). | | Effective for PA promotion. | |  |
| Mamede [48] | 2-arm RCT; IG: gamified digital app, CG: active control. | | Those in the intervention receiving the full version of the digital app, MoveMore, consisted of a 5-week gamification phase that included a commercially available gamified digital app incorporating several BCTs and nudges, such as social support and social comparison, followed by a physical nudges phase for the last 5 weeks. | | Behavior change techniques | | 5 | | Application and wearable device. | | Daily step counts by accelerometer:  IG: compared with CG, increased 634 (*P*=.01). | | No significant changes were observed in hours in light PA, MVPA, and sitting. | | Effective for PA promotion. | |  |
| Monroe [49] | 2-arm RCT; IG: Technology-delivered PA intervention (TECH)+gamification, CG: TECH. | | The TECH+Gamifcation group also engaged with custom-designed gamifcation elements underpinned by the SCT, self-determination theory, social network theory, and behavioral economics principles. | | Social cognitive theory, self-determination theory, social network theory, and behavioral economics principles | | 12 | | Application and wearable device. | | Daily step countsby accelerometer:  IG-CG: decreased 425 steps (*P*=.19). | | MVPA by accelerometer:  IG-CG: decreased 3.36 min/day (*P*=.21). | | Not effective for PA promotion. | |  |
| Nishiwaki [50] | 2-arm crossover RCT; IG: game intervention, CG: normal activity monitor. | | The participants wore both a normal activity monitor and an activity monitor with computerized game functions during the game intervention period. | | / | | 12 | | Activity monitor with game function. | | Daily step counts by activity monitor:  IG vs CG: 10,520 ± 562 vs 8,711±523 (*P*<.01). | | Amount of PA (METs h/day) by activity monitor:  IG vs CG: 3.1±0.2 vs 2.4±0.2 (*P*<.01). | | Effective for PA promotion. | |  |
| Nuijten [51] | 2-arm RCT; IG: personalized complexity parameters, CG: general complexity parameters. | | GameBus was especially designed for health promotion and provides a highly configurable gamification engine that is used for sustaining participant engagement. | | / | | 8 | | Application. | | Personalization was effective for promoting physical activity frequency. | | / | | Personalization seems particularly promising for promoting the frequency of physical activity. | |  |
| Patel [53] | 2-arm RCT; IG: gamification intervention plus daily feedback, CG: daily feedback control. | | Participants in the gamification arm were entered into a game with their family for 12 weeks that was designed using insights from behavioral economics to address predictable barriers to behavior change and to enhance social incentives. | | behavioral economics principles | | 12 | | Application, text messages and wearable device. | | Daily step counts:  IG-CG: MD 953 steps/day, (*P*<.001). | | Proportion of days meeting their step goal:  IG-CG: Increased 0.27 participant-day (*P*<.001). | | Effective for PA promotion. | |  |
| Patel [54] | 4-arm RCT; IG1: support gamification, IG2: collaboration gamification, IG3: competition gamification, CG: regular feedback control. | | Participants in the intervention arms were entered into a game with points and levels that was run automatically and provided a daily notification of their progress. | | behavioral economics principles | | 24 | | Application, text messages and wearable device. | | Daily step counts:  IG1-CG: MD 689 steps/day (*P*<.001);  IG2-CG: MD 637 steps/day (*P*=.001);  IG3-CG: MD 920 steps/day (*P*<.001). | | Proportion of days meeting their step goal:  IG1-CG: Increased 0.11 participant-day (*P*<.001);  IG2-CG: Increased 0.11 participant-day (*P*<.001);  IG3-CG: Increased 0.16 participant-day (*P*<.001). | | Effective for PA promotion. | |  |
| Patel [52] | 5-arm RCT; IG1: gamification with assigned and immediate goals, IG2: gamification with assigned and gradual goals, IG3: gamification with choice and immediate goals, IG4: gamification with choice and gradual goals, CG: daily feedback control. | | Participants in the gamification arms were randomly assigned to have step goals either assigned or self-chosen. Participants in the self-chosen arms could change their goal at any time during the study as long as it was within the provided range. When the study began, goals were implemented either immediately or gradually over the 8-week introductory period. | | behavioral economics principles | | 16 | | Application, text messages and wearable device. | | Daily step counts at week 9 to 16:  IG1-CG: MD 523 steps/day (*P*=.08);  IG2-CG: MD 616 steps/day (*P*=.04);  IG3-CG: MD 1384 steps/day (*P*<.001);  IG4-CG: MD 569 steps/day (*P*=.05). | | Daily step counts at week 17 to 24:  IG1-CG: MD 473 steps/day (*P*=.12);  IG2-CG: MD 776 steps/day (*P*=.01);  IG3-CG: MD 1391 steps/day (*P*<.001);  IG4-CG: MD 578 steps/day (*P*=.06). | | Effective for PA promotion. | |  |
| Patel [55] | 4-arm RCT; IG1: support gamification, IG2: collaboration gamification, IG3: competition gamification, CG: goal setting and regular feedback control. | | Participants in the intervention arms were entered into a game with points and levels that ran automatically and provided a daily notification on their progress. | | behavioral economics principles | | 48 | | Application, text messages and wearable device. | | Daily step counts:  IG1-CG: MD 503 steps/day (*P*=.01);  IG2-CG: MD 280 steps/day (*P*=.16);  IG3-CG: MD 606 steps/day (*P*=.003). | | / | | Effective for PA promotion for support and competition group. | |  |
| Pyky [56] | 2-arm RCT; IG: MOPOrtal service, CG: received wearable device. | | The intervention group was given access to a novel mobile service (MOPOrtal). The aim of the MOPOrtal was to motivate participants physically, mentally and socially. In addition, the participants in the intervention group received tailored feedback. | | Transtheoretical model | | 24 | | Web and  wearable device. | | Life satisfaction:  IG: improved (*P*<.001), CG: improved (*P*=.01). | | No significant changes were observed in self-rated health. | | Effective for life satisfaction improvement. | |  |
| Tu [64] | 2-arm RCT; IG1: Walkup app, IG2: WeChat Sports app. | | Walkup and WeChat Sports were selected because these two fitness apps are distinct enough to represent different gamification orientations: Walkup includes five commonly used game elements that aim at enhancing enjoyment, and has few social interaction elements; in contrast, WeChat Sport includes three game elements that are commonly applied to enhance social interaction, but has none of the enjoyment-enhancing elements that Walkup has. | | / | | 7 | | Application. | | Daily step counts on the last day (mean±SD):  IG1: Walkup 4737.32±5446.46;  IG2: WeChat Sports 8775.80±5421.74. | | Intentions to continue exercising (mean±SD):  IG1: 2.97±1.11;  IG2: 3.76±0.81. | | Compared to make it fun, making it more social can be more effective in helping  them sustain a walking routine in their daily living. | |  |
| Robertson [57] | 2-arm RCT; IG: FitQuest app, CG: standard PE lesson. | | The most recent version of the system runs on the Android operating system, utilising GPS technology and Google MapsTM in order to provide a series of 8 exercise based mini-games. | | / | | 5 | | Application. | | Self-efficacy by scale:  IG: from 24.7±4.1 to 25.8 ±4.1;  CG: from 24.9±4.4 to 24.8±4.1. | | Step count:  IG: from 6033±1809 to 5213±1692;  CG: from 6287±1414 to 6081±1945. | | Not effective for PA promotion. | |  |
| Robertson [58] | 2-arm RCT; IG: multimedia messaging service messages plus activity tracker, CG: received physical activity tracker. | | The participants in the experimental group received Steps2Health multimedia messaging service (MMS) messages. These messages presented information about a virtual journey through Japan’s Inland Sea region and were triggered by step counts in real time. | | Self-determination theory | | 4 | | Web, application and wearable device. | | Motivation for physical activity:  Both groups improved (*P*=.04). | | - Self-reported leisure activity score: IG improved (*P*=.005); - Daily step counts: IG improved (*P*=.02). | | Effective for PA promotion. | |  |
| Santos [59] | 2-arm RCT; IG: social pervasive game, CG: pervasive game without social interaction. | | Participants played a pervasive location-based mobile game called Shinpo, which, in free translation, means “steps of the gods” in Japanese. | | / | | 4 | | Application. | | Weekly step counts changes to baseline values (mean±SE):  IG: 7648±3900.9;  CG: -1041.8±1992.7. | | / | | Effective for PA promotion. | |  |
| Sanudo [60] | 2-arm RCT; IG: gamified mHealth intervention, CG: control condition without gamification. | | Study was designed with a specific aim to examine the effects of gamification by modifying social comparison. Additionally, it is essential to recognize that the intervention also inherently incorporated other behavior change techniques, grounded in established theories. | | Behavior change techniques | | 8 | | Application and wearable device. | | - Step counts by accelerometer: IG: from 8278.8±4073.8 to 11895.7±4976.0, *P*<0.001; CG: from 7580.0±3937.6 to 12203±4711.6, *P*<0.001. - MVPA (min/weeks) by accelerometer: IG: from 119.6±56 to 164.4±79.3, *P*=0.005; CG: from 98.7±50.4 to 163.3±61.5, *P*<0.001. | | Sleep hours (h/day) by accelerometer:  IG: from 7.6±1.0 to 7.8±1.0, *P*=0.56;  CG: from 7.7±1.6 to 7.5±1.9, *P*=0.54. | | Not effective for PA promotion regarding the addition of gamified social comparison. | |  |
| Schwarz [61] | 2-arm RCT; IG: dynamically tailored exergame, CG: non-tailored exergame. | | The study is part of the European SmartLife project which aims to increase physical activity among adolescents using a mobile exergame. The game developed for this project consisted of a narrative-driven, single player, audio-supported mobile exergame. | | / | | 4 | | Application and  accelerometer T-shirt. | | Overall kids game experience questionnaire:  IG: 2.03; CG: 1.97, *P*=0.62. | | / | | Not effective for game engagement improvement. | |  |
| Simmering [62] | 2-arm RCT; IG: VA MapTrek, CG: received a Fitbit activity tracker. | | Half of the veterans were assigned to participate in VA MapTrek, a text-message-based race game designed to increase step counts. | | / | | 8 | | Application and wearable device. | | Step counts by fitbit:  IG have taken an extra 96,627 steps (equivalent to 48.3 additional miles) than CG. | | / | | Effective for PA promotion. | |  |
| Thorsteinsen [63] | 2-arm RCT; IG: Lifestyle intervention, CG: no intervention. | | The intervention Lifestyle Tool consisted of a rule-based website designed to help people plan and monitor their physical activity in order to become more physically active. | | / | | 12 | | Web. | | - Minutes exercising at week 5: IG: 576.67±235.29, CG: 413.13±224.42; - Minutes exercising at week 9: IG: 660.25±359.60, CG: 377.25±167.39; - Minutes exercising at week 12: IG: 574.42±434.74, CG: 501.88±232.90. | | / | | Effective for PA promotion at a short-term. | |  |
| Waddell [65] | 2-arm RCT; IG: gamification with social incentives, CG: received wearable device feedback. | | The gamification arm engaged in an 8-week game with loss-framed points and levels to help achieve their step goal and received daily (text message) and weekly (email) feedback describing their progress. | | behavioral economics principles | | 8 | | Text messages and  wearable device. | | Daily step counts:  IG-CG: MD 981 steps/day, *P*=.01. | | Proportion of days meeting their step goal:  IG-CG: Increased 0.41 participant-day (*P*<.001). | | Effective for PA promotion. | |  |
| Xu [66] | 3-arm RCT; IG1: individual gamified intervention, IG2: team gamified intervention, CG: received daily goal setting. | | Patients in the individual and team groups received the  gamification intervention based on behavioral economics  principles via the WeChat applet. | | behavioral economics principles | | 12 | | Application. | | Daily step counts:  IG1-CG: MD 988 steps/day, *P*<.01;  IG2-CG: MD 472 steps/day, *P*=.12. | | Proportion of days meeting their step goal:  IG1-CG: Increased 0.08 participant-day (*P*<.001);  IG2-CG: Increased 0.04 participant-day (*P*=.074). | | Effective for PA promotion in IG1. | |  |
| Zuckerman [67] | 3-arm RCT; IG1: points version of StepByStep, IG2: leaderboard version, CG: quantified version. | | The study evaluated the effectiveness of two game elements: virtual rewards and social comparison. | | / | | 2 | | Application. | | Daily walking time:  IG1: 49.94±24.78, IG2: 40.85±18.40, CG: 43.63±20.36. | | / | | Effective for PA promotion in points version. | |  |
| Maria do [74] | 3-arm RCT; IG1: smartphone app, IG2: smartphone app+ tailored messages, IG3: smartphone+tailored messages+gamification, CG: usual routine. | | The intervention involves step monitoring, individual messages, and virtual challenges. For each challenge they completed, they were rewarded with a virtual badge on the app. There were rankings of the number of steps among all users, as well as running challenges. | | / | | 24 | | Application. | | Daily step counts by self-report: IG: 3995 ± 3204 vs CG: 4250 ± 4204. | | Steps per day after rerandomization: median -198 to +20 steps/day. | | The SMART design was feasible and changed the behavior of steps per day after rerandomization. | |  |
| Patel [68] | 2-arm RCT; IG: gamification+automated coaching, CG: attention control. | | All participants received a Fitbit, selected a step-count goal, and were advised to undertake home-based walking. IG participants entered a 16-week automated loss-framed game with weekly points, 5 levels, fresh starts, and a support partner; they also received biweekly automated coaching text messages. | | Behavioral economics principles | | 16 | | Text messages and wearable device. | | Daily step counts during weeks 5-16: IG-CG: +920 steps/day (95% CI -21 to 1861; P=.06). | | Daily step counts during weeks 17-24: IG-CG: +1074 steps/day (95% CI 133 to 2015; *P*=.03). | | Gamification plus automated coaching increased PA compared with attention control over the 24-week study, with a significant effect during follow-up. | |  |
| Patel [69] | 2-arm RCT; IG: behaviorally designed gamification, CG: attention control. | | All participants received a Fitbit, established a baseline step count, and selected a step goal increase. IG participants entered a 24-week automated game using a precommitment pledge, weekly loss-framed points, levels, fresh starts, a support partner, and a nominal trophy. | | Behavioral economics principles | | 24 | | Text messages and wearable device. | | Daily step counts during the 24-week intervention: IG-CG: +759 steps/day (95% CI 209 to 1309; *P*=.007). | | Weekly MVPA during intervention: IG-CG +16 min/week (95% CI 4 to 29; *P*=.010); during 12-week follow-up: +11 min/week (95% CI 0 to 22; *P*=.048). Daily steps during follow-up: +581 steps/day (*P*=.070). | | Behaviorally designed gamification increased PA among Black and Hispanic breast and prostate cancer survivors. | |  |
| Janis [70] | 2-arm cluster RCT; IG: SMARTFAMILY2.0 app, CG: waiting-list control. | | Families received study smartphones with the SMARTFAMILY2.0 app and Bluetooth-linked accelerometers. They set collective weekly goals for steps, MVPA, joint family PA, fruit and vegetable intake, and shared meals. The app provided an animated tailored coach, points/stars and badges, personalized progress feedback, leaderboards and peer support, weekly goal prompts, and just-in-time prompts after prolonged inactivity. | | Self-determination theory and behavior change techniques; just-in-time adaptive intervention. | | 3 | | Application and wearable device. | | Primary outcomes: Device-measured MVPA favored the CG at T1 (β = −.50, *P*=.02); no significant effects were found for step count or self-reported MVPA. | | Secondary outcomes: Joint PA favored the IG at T1 and T2. | | Not effective for increasing overall PA in an already active sample. | |  |
| Nimet [71] | 2-arm single-blind cluster RCT; IG: gamified T2M Android mobile application; CG: nongamified T2M Android mobile application. | | Students used the T2M Android app to pursue a daily goal of 10,000 steps and complete exercise goals. Daily step counts were measured with a pedometer and uploaded to the app, while exercise videos were viewed and participants recorded their exercise performance. The IG additionally received points, digital step and exercise badges, a leaderboard, and achievement certificates and medals. | | Self-determination theory. | | 4 | | Application and pedometer. | | Daily goal completion favored the IG: step-goal completion was 65% versus 40% in the CG (*P*=.043, d=.40), and exercise-goal completion was 68% versus 15% (*P*=.0001, d=.77). | | PAQ-C scores improved more in the IG than in the CG (*P*=.0114, d=.51). At 6 months, 70% of the IG versus 27% of the CG reported continuing to exercise. | | The gamified T2M app increased completion of daily step and exercise goals and improved self-reported physical activity among sedentary secondary school students, with some maintenance of exercise behavior at 6 months. | |  |
| Gaizka [72] | 2-arm single-center pragmatic RCT; IG: gamified family-based online exercise platform; CG: routine care. | | Children completed a 12-week family-based exercise program through an online gamified platform at home or outdoors, with parental supervision. Two sessions were available each week and included warm-up, bodyweight strength, cardiorespiratory, color-coded, and cooldown exercises. Exercises increased progressively in length and intensity. The platform included points, a calendar/session guide, customizable avatars, 14 unlockable worlds, novice-to-expert levels, virtual accessories and other milestone-contingent rewards. Platform use and session completion were monitored weekly, and nonadherent families received telephone encouragement and support. | | / | | 12 | | Web-based online gamified exercise platform. | | No significant between-group effect was found for accelerometer-measured MVPA after 12 weeks. | | The IG showed greater improvements in light PA (+26.44 min/day, *P*=.01), total PA (+30.88 min/day, *P*=.02), standing long jump (+12.26 cm, *P*<.001), and overall physical fitness z-score (+0.15, *P*=.04). Other movement, fitness, motor competence, and executive-function outcomes did not differ significantly. | | The gamified family-based intervention improved light and total physical activity and selected physical fitness outcomes, but did not significantly improve the primary MVPA outcome. | |  |
| Jeffrey Z [75] | 4-arm parallel RCT; SG: gamification elements introduced one at a time; CG: the same elements introduced sequentially and retained cumulatively; EG: commercial mobile walking game; control: tracking only. | | All intervention groups selected an individualized daily step goal. SG received, in sequence, points with weekly progress feedback, daily leaderboards with recognition of the top 3 participants, team-based social interaction and step-count competition through LINE OpenChat, and Pikmin Bloom. CG received the same elements, but each remained active after introduction. EG played Pikmin Bloom throughout the intervention; the game linked real-world steps to points and levels and included avatars, virtual character collection, location-based exploration, social collaboration, and community challenges. | | / | | 7 | | Applications. | | Daily step count by smartphone app-based self-report: all gamification arms increased steps versus control during intervention; cumulative delivery produced the largest effect (+2,326 steps/day, *P*<.001). At follow-up, effects remained significant for cumulative delivery (+1,284/day, *P*=.005) and explicit gamification (+1,027/day, *P*=.025), but not sequential delivery. | | No consistent biometric effects were found; resting heart rate improved transiently in SG and systolic blood pressure improved transiently in CG, but these effects were not maintained at follow-up. | | Cumulative delivery of gamification elements was the most effective strategy for increasing daily steps. Explicit gamification showed a slower but more durable pattern, whereas the effect of sequential delivery was not sustained after intervention withdrawal. | |  |
| Alexandre [73] | 2-arm parallel RCT; IG: Kiplin digital gamified adapted physical activity program; CG: usual-care face-to-face supervised adapted physical activity program. | | The 12-week Kiplin intervention comprised 2 initial face-to-face sessions, 20 live group telecoaching sessions, 3 collective mobile-app games, an in-app chat, and real-time activity monitoring linked to a Garmin wearable. Daily steps were converted into points and used to advance through game narratives: collectively reaching destinations on a world map, completing challenges to unlock clues and solve missions, and progressing through levels in a forest-rescue board game. | | Social identity approach and self-determination theory. | | 12 | | Application and wearable device. | | Wearable-measured daily steps: compared with usual care, the Kiplin group showed a greater increase from baseline during the 3-month intervention (+1085 steps/day; 95% CI 493-1676; *P*<.001) and the 6-month postintervention follow-up (+1775 steps/day; 95% CI 906-2643; *P*<.001). | | MVPA improved in favor of the Kiplin group (group×time β=5.69, 95% CI 0.61-10.77; *P*=.03). No significant group×time effects were found for the other clinical outcomes, and cost-utility did not differ significantly between programs. | | The digital gamified Kiplin program increased and sustained daily physical activity compared with usual care, although responses varied substantially between individuals and no clear clinical or economic superiority was demonstrated. | |  |
| B. Sañudo [76] | 2-arm multicentre pilot RCT; IG: IDHEApp gamified mHealth intervention plus Fitbit Charge 6; CG: usual routine with Fitbit Charge 6 used for measurement only. | | The 8-week IDHEApp intervention used Fitbit Charge 6 data and delivered daily personalised challenges targeting physical activity, sedentary behavior, and nutrition. Participants earned points, received digital badges for behavioral milestones, viewed visual progress indicators, received notifications and feedback, and could access overall and behavior-specific leaderboards. | | / | | 8 | | Application and wearable device. | | Daily steps: the intervention group had a significantly greater post-intervention step count than the control group after baseline adjustment (adjusted mean difference 2326.72 steps/day, 95% CI 763.97-3461.23; *P*=.001; Cohen d=0.97). | | No significant between-group effects were found for light, moderate, or vigorous physical activity, sitting time, or standing time. | | The pilot RCT suggests that an accessible gamified mHealth intervention is feasible and may increase daily ambulatory activity among young people with intellectual disabilities, although evidence for other activity intensities and sedentary behavior remained exploratory. | |  |
| Ming [77] | 2-arm parallel-group RCT; IG: gamified team-based mHealth intervention; CG: active technology-based PA monitoring. | | Both groups used the Shouti Fitness app and a fitness watch, with daily targets of ≥30 min MVPA or ≥180 MET-min and weekly targets of ≥150 min MVPA or ≥900 MET-min. The IG was additionally assigned to 4 teams and received rank-based points, top-performer bonus points, real-time MVPA/MET-min progress bars, midweek champion updates, weekly team and individual leaderboards, cumulative volunteer hours, honorary certificates, academic-credit coordination, and a 400-Chinese-yuan material reward for top-10% performers. | | Self-determination theory and theory of planned behavior. | | 8 | | Application and wearable device. | | Across 8 weeks, IG versus CG: daily steps 10,356±1,245 versus 8,242±1,087 (Δ=2,114; d=1.81); daily MVPA 71±15 versus 43±12 min (Δ=28; d=2.06); weekly MET-min 1,650±310 versus 1,340±285 (Δ=310; d=1.04); goal completion 88.9%±8.5 versus 77.5%±10.2 and data synchronization 89.1%±7.7 versus 75.5%±11.3 (all *P*<.001). | | Significant effects favored the IG for skeletal muscle (+0.54 kg), body fat (−0.46 percentage points), basal metabolic rate (+7.8 kcal/day), 800/1000-m run time (−6.5 s), vital capacity (+190 mL), sit-and-reach (+1.8 cm), female sit-ups (+4.5), cognitive flexibility (−29 ms), and depressive symptoms (−2.0 points). | | Adding team-based gamification and incentives to an otherwise identical wearable-linked mHealth PA platform substantially improved PA, adherence, and selected physical, cognitive, and mental health outcomes among college students; no serious adverse events occurred. | |  |

REFERENCES

1. 25. Agarwal AK, Waddell KJ, Small DS, et al. Effect of gamification with and without financial incentives to increase physical activity among veterans classified as having obesity or overweight: a randomized clinical trial. JAMA Netw Open. Jul 1, 2021;4(7):e2116256. [doi: ] [Medline: 34241628]
2. 26. Corepal R, Best P, O’Neill R, et al. A feasibility study of “The StepSmart Challenge” to promote physical activity in adolescents. Pilot Feasibility Stud. 2019;5(132):132. [doi: ] [Medline: 31832227]
3. 27. Dadaczynski K, Schiemann S, Backhaus O. Promoting physical activity in worksite settings: results of a German pilot study of the online intervention Healingo fit. BMC Public Health. Sep 8, 2017;17(1):696. [doi: ] [Medline: 28886734]
4. 28. Direito A, Jiang Y, Whittaker R, Maddison R. Apps for IMproving FITness and increasing physical activity among young people: the AIMFIT pragmatic randomized controlled trial. J Med Internet Res. Aug 27, 2015;17(8):e210. [doi: ] [Medline: 26316499]
5. 29. Edney S, Ryan JC, Olds T, et al. User engagement and attrition in an app-based physical activity intervention: secondary analysis of a randomized controlled trial. J Med Internet Res. Nov 27, 2019;21(11):e14645. [doi: ] [Medline: 31774402]
6. 30. Fanaroff AC, Patel MS, Chokshi N, et al. Effect of gamification, financial incentives, or both to increase physical activity among patients at high risk of cardiovascular events: the BE ACTIVE randomized controlled trial. Circulation. May 21, 2024;149(21):1639-1649. [doi: ] [Medline: 38583084]
7. 31. Francis SL, Simmering JE, Polgreen LA, et al. Gamifying accelerometer use increases physical activity levels of individuals pre-disposed to type II diabetes. Prev Med Rep. Sep 2021;23:101426. [doi: ] [Medline: 34178586]
8. 32. Garde A, Chowdhury M, Rollinson AU, et al. A 0multi-week assessment of a mobile exergame intervention in an elementary school. Games Health J. Feb 2018;7(1):1-8. [doi: ] [Medline: 29394109]
9. 33. Garde A, Umedaly A, Abulnaga SM, et al. Evaluation of a novel mobile exergame in a school-based environment. Cyberpsychol Behav Soc Netw. Mar 2016;19(3):186-192. [doi: ] [Medline: 26882222]
10. 34. Gillcrist RL, Doherty CR, Olave M, et al. A remote behaviorally designed intervention to promote physical activity in patients with knee osteoarthritis: results of a pilot randomized clinical trial. J Clin Rheumatol. Dec 1, 2024;30(8):336-339. [doi: ] [Medline: 39412672]
11. 35. Gonze BDB, Padovani RDC, Simoes MDS, et al. Use of a smartphone app to increase physical activity levels in insufficiently active adults: feasibility sequential multiple assignment randomized trial (SMART). JMIR Res Protoc. Oct 23, 2020;9(10):e14322. [doi: ] [Medline: 33094733]
12. 36. Gremaud AL, Carr LJ, Simmering JE, et al. Gamifying accelerometer use increases physical activity levels of sedentary office workers. J Am Heart Assoc. Jul 2, 2018;7(13):e007735. [doi: ] [Medline: 29967221]
13. 37. Greysen SR, Changolkar S, Small DS, et al. Effect of behaviorally designed gamification with a social support partner to increase mobility after hospital discharge. JAMA Netw Open. Mar 1, 2021;4(3):e210952. [doi: ] [Medline: 33760089]
14. 38. Greysen SR, Oon AL, Harkins K, et al. Effect of gamification with a support partner to increase physical activity in older adults at risk for Alzheimer’s disease: the STEP 4Life randomized clinical trial. Alzheimers Dement. Aug 2024;20(8):5450-5459. [doi: ] [Medline: 38962958]
15. 39. Haque MS, Kangas M, Jämsä T. A persuasive mHealth behavioral change intervention for promoting physical activity in the workplace: feasibility randomized controlled trial. JMIR Form Res. May 4, 2020;4(5):e15083. [doi: ] [Medline: 32364506]
16. 40. Höchsmann C, Infanger D, Klenk C, Königstein K, Walz SP, Schmidt-Trucksäss A. Effectiveness of a behavior change technique-based smartphone game to improve intrinsic motivation and physical activity adherence in patients with type 2 diabetes: randomized controlled trial. JMIR Serious Games. Feb 13, 2019;7(1):e11444. [doi: ] [Medline: 30758293]
17. 41. Höchsmann C, Müller O, Ambühl M, et al. Novel smartphone game improves physical activity behavior in type 2 diabetes. Am J Prev Med. Jul 2019;57(1):41-50. [doi: ] [Medline: 31128953]
18. 42. Kurtzman GW, Day SC, Small DS, et al. Social incentives and gamification to promote weight loss: the LOSE IT randomized, controlled trial. J Gen Intern Med. Oct 2018;33(10):1669-1675. [doi: ] [Medline: 30003481]
19. 43. Lee D, Frey GC, Cothran DJ, Harezlak J, Shih PC. Effects of a gamified, behavior change technique-based mobile app on increasing physical activity and reducing anxiety in adults with autism spectrum disorder: feasibility randomized controlled trial. JMIR Form Res. Jul 28, 2022;6(7):e35701. [doi: ] [Medline: 35900808]
20. 44. Leinonen AM, Pyky R, Ahola R, et al. Feasibility of gamified mobile service aimed at physical activation in young men: population-based randomized controlled study (MOPO). JMIR Mhealth Uhealth. Oct 10, 2017;5(10):e146. [doi: ] [Medline: 29017991]
21. 45. Lewey J, Murphy S, Zhang D, et al. Effectiveness of a text-based gamification intervention to improve physical activity among postpartum individuals with hypertensive disorders of pregnancy: a randomized clinical trial. JAMA Cardiol. Jun 1, 2022;7(6):591-599. [doi: ] [Medline: 35442393]
22. 46. Lin PJ, Fanjiang YY, Wang JK, et al. Long-term effectiveness of an mHealth-tailored physical activity intervention in youth with congenital heart disease: a randomized controlled trial. J Adv Nurs. Aug 2021;77(8):3494-3506. [doi: ] [Medline: 34151444]
23. 47. Maher C, Ferguson M, Vandelanotte C, et al. A web-based, social networking physical activity intervention for insufficiently active adults delivered via facebook app: randomized controlled trial. J Med Internet Res. Jul 13, 2015;17(7):e174. [doi: ] [Medline: 26169067]
24. 48. Mamede A, Noordzij G, Jongerling J, Snijders M, Schop-Etman A, Denktas S. Combining web-based gamification and physical nudges with an app (MoveMore) to promote walking breaks and reduce sedentary behavior of office workers: field study. J Med Internet Res. Apr 12, 2021;23(4):e19875. [doi: ] [Medline: 33843593]
25. 49. Monroe CM, Cai B, Edney S, et al. Harnessing technology and gamification to increase adult physical activity: a cluster randomized controlled trial of the Columbia Moves pilot. Int J Behav Nutr Phys Act. Nov 3, 2023;20(1):129. [doi: ] [Medline: 37924083]
26. 50. Nishiwaki M, Kuriyama A, Ikegami Y, Nakashima N, Matsumoto N. A pilot crossover study: effects of an intervention using an activity monitor with computerized game functions on physical activity and body composition. J Physiol Anthropol. Dec 2, 2014;33(1):35. [doi: ] [Medline: 25466450]
27. 51. Nuijten R, Van Gorp P, Khanshan A, et al. Evaluating the impact of adaptive personalized goal setting on engagement levels of government staff with a gamified mHealth tool: results from a 2-month randomized controlled trial. JMIR Mhealth Uhealth. Mar 31, 2022;10(3):e28801. [doi: ] [Medline: 35357323]
28. 52. Patel MS, Bachireddy C, Small DS, et al. Effect of goal-setting approaches within a gamification intervention to increase physical activity among economically disadvantaged adults at elevated risk for major adverse cardiovascular events: the ENGAGE randomized clinical trial. JAMA Cardiol. Dec 1, 2021;6(12):1387-1396. [doi: ] [Medline: 34468691]
29. 53. Patel MS, Benjamin EJ, Volpp KG, et al. Effect of a game-based intervention designed to enhance social incentives to increase physical activity among families: the BE FIT randomized clinical trial. JAMA Intern Med. Nov 1, 2017;177(11):1586-1593. [doi: ] [Medline: 28973115]
30. 54. Patel MS, Small DS, Harrison JD, et al. Effectiveness of behaviorally designed gamification interventions with social incentives for increasing physical activity among overweight and obese adults across the United States: the STEP UP randomized clinical trial. JAMA Intern Med. Dec 1, 2019;179(12):1624-1632. [doi: ] [Medline: 31498375]
31. 55. Patel MS, Small DS, Harrison JD, et al. Effect of behaviorally designed gamification with social incentives on lifestyle modification among adults with uncontrolled diabetes: a randomized clinical trial. JAMA Netw Open. May 3, 2021;4(5):e2110255. [doi: ] [Medline: 34028550]
32. 56. Pyky R, Koivumaa-Honkanen H, Leinonen AM, et al. Effect of tailored, gamified, mobile physical activity intervention on life satisfaction and self-rated health in young adolescent men: a population-based, randomized controlled trial (MOPO study). Comput Human Behav. Jul 2017;72:13-22. [doi: ]
33. 57. Robertson J, Macvean A, Fawkner S, Baker G, Jepson RG. Savouring our mistakes: learning from the FitQuest project. Int J Child Comput Interact. Jun 2018;16:55-67. [doi: ]
34. 58. Robertson MC, Lyons EJ, Liao Y, Baum ML, Basen-Engquist KM. Gamified text messaging contingent on device-measured steps: randomized feasibility study of a physical activity intervention for cancer survivors. JMIR Mhealth Uhealth. Nov 24, 2020;8(11):e18364. [doi: ] [Medline: 33231551]
35. 59. Santos L, Okamoto K, Otsuki R, et al. Promoting physical activity in Japanese older adults using a social pervasive game: randomized controlled trial. JMIR Serious Games. Jan 6, 2021;9(1):e16458. [doi: ] [Medline: 33404507]
36. 60. Sañudo B, Sanchez-Trigo H, Domínguez R, et al. A randomized controlled mHealth trial that evaluates social comparison-oriented gamification to improve physical activity, sleep quantity, and quality of life in young adults. Psychol Sport Exerc. May 2024;72:102590. [doi: ] [Medline: 38218327]
37. 61. Schwarz A, Cardon G, Chastin S, et al. Does dynamic tailoring of a narrative-driven exergame result in higher user engagement among adolescents? Results from a cluster-randomized controlled trial. Int J Environ Res Public Health. Jul 12, 2021;18(14):7444. [doi: ] [Medline: 34299895]
38. 62. Simmering JE, Polgreen LA, Francis SL, Strom AJ, Segre AM, Polgreen PM. Using a Fitbit-based walking game to improve physical activity among U.S. veterans. Mil Med. Jan 16, 2025;190(1-2):194-201. [doi: ] [Medline: 38829720]
39. 63. Thorsteinsen K, Vittersø J, Svendsen GB. Increasing physical activity efficiently: an experimental pilot study of a website and mobile phone intervention. Int J Telemed Appl. 2014;2014:746232. [doi: ] [Medline: 24963290]
40. 64. Tu R, Hsieh P, Feng W. Walking for fun or for “likes”? The impacts of different gamification orientations of fitness apps on consumers’ physical activities. Sport Manag Rev. Dec 1, 2019;22(5):682-693. [doi: ]
41. 65. Waddell KJ, Patel MS, Clark K, Harrington TO, Greysen SR. Effect of gamification with social incentives on daily steps after stroke: a randomized clinical trial. JAMA Neurol. May 1, 2022;79(5):528-530. [doi: ] [Medline: 35344027]
42. 66. Xu L, Tong Q, Zhang X, et al. Smartphone-based gamification intervention to increase physical activity participation among patients with coronary heart disease: a randomized controlled trial. J Telemed Telecare. Oct 2024;30(9):1425-1436. [doi: ] [Medline: 36794484]
43. 67. Zuckerman O, Gal-Oz A. Deconstructing gamification: evaluating the effectiveness of continuous measurement, virtual rewards, and social comparison for promoting physical activity. Pers Ubiquit Comput. Oct 2014;18(7):1705-1719. [doi: ]
44. 68. Fanaroff AC, Coratti S, Farraday D, et al. Effect of gamification plus automated coaching to increase physical activity among patients with peripheral artery disease: the GAMEPAD randomized controlled trial. J Am Heart Assoc. Dec 16, 2025;14(24):e038921. [doi: ] [Medline: 41334739]
45. 69. Fanaroff AC, Orr JA, Anucha C, et al. Behaviorally designed gamification and physical activity among breast and prostate cancer survivors. JACC CardioOncol. Dec 2025;7(7):816-828. [doi: ] [Medline: 41182202]
46. 70. Fiedler J, Wunsch K, Hubenschmid S, Reiterer H, Renner B, Woll A. A refined mobile health intervention (SMARTFAMILY2.0) to promote physical activity and healthy eating in a family setting: randomized controlled trial. JMIR Mhealth Uhealth. Dec 15, 2025;13:e65558. [doi: ] [Medline: 41397249]
47. 71. Haşıl Korkmaz N, Çoruh H. Time to move: a 4-week gamified mobile application intervention to promote physical activity in secondary school students. Eur J Sport Sci. Aug 2025;25(8):e70027. [doi: ] [Medline: 40712065]
48. 72. Legarra-Gorgoñon G, García-Alonso Y, Ramírez-Vélez R, Alonso-Martínez L, Izquierdo M, Alonso-Martínez AM. Effect of a gamified family-based exercise intervention on adherence to 24-hour movement behavior recommendations in preschool children: single-center pragmatic trial. JMIR Serious Games. Mar 4, 2025;13:e60185. [doi: ] [Medline: 40036649]
49. 73. Mazéas A, Chalabaev A, Blond M, Mourgues C, Pereira B, Duclos M. Effect of a digital health physical activity program integrating gamification for obesity management in comparison with usual care: randomized controlled trial with an ideographic approach. J Med Internet Res. Nov 28, 2025;27:e78376. [doi: ] [Medline: 41328457]
50. 74. Simoes M do S, Proença NL, Lauria VT, do Nascimento MB, Padovani R da C, Dourado VZ. Effects of using a smartphone app combined with behavior change techniques on the level of physical activity among adults and older adults: sequential multiple assignment randomized trial. J Med Internet Res. Jan 30, 2026;28:e73388. [doi: ] [Medline: 41616115]
51. 75. Liu JZ, Chen RY, Rouyard T, Munkhtogoo D, Tsai FJ. Effects of different gamification delivery strategies on physical activity in young adults: an RCT. Am J Prev Med. Jul 2026;71(1):108297. [doi: ] [Medline: 41655647]
52. 76. Sañudo B, Sañudo JI, Feria A, Schwarz D, Langbein E, Sánchez-Oliver AJ. Promoting healthy behaviours in young people with intellectual disabilities: a pilot RCT of an mHealth-based intervention targeting physical activity and sedentary behaviour. Disabil Rehabil Assist Technol. May 15, 2026;15(1-13):1-13. [doi: ] [Medline: 42138210]
53. 77. Yang M, Guo Y, Li Z, et al. A gamified mobile health intervention to promote physical activity, executive function, and mental health in college students: randomized controlled trial. J Med Internet Res. Apr 7, 2026;28:e82769. [doi: ] [Medline: 41945642]
